# Influence of Solvent on Selective Catalytic Reduction of Nitrogen Oxides with Ammonia over Cu-CHA Zeolite

**DOI:** 10.1021/jacs.2c09823

**Published:** 2022-12-22

**Authors:** Jamal Abdul Nasir, Jingcheng Guan, Thomas W. Keal, Alec W. Desmoutier, You Lu, Andrew M. Beale, C. Richard A. Catlow, Alexey A. Sokol

**Affiliations:** † Department of Chemistry, Kathleen Lonsdale Materials Chemistry, University College London, 20 Gordon Street, LondonWC1H 0AJ, U.K.; # Department of Chemistry, Christopher Ingold Building, University College London, 20 Gordon Street, LondonWC1H 0AJ, U.K.; ‡ UK Catalysis Hub, Research Complex at Harwell, Rutherford Appleton Laboratory, R92 Harwell, OxfordshireOX11 0FA, U.K.; § School of Chemistry, Cardiff University, Park Place, CardiffCF10 3AT, U.K.; ∥ Scientific Computing Department, STFC Daresbury Laboratory, Keckwick Lane, Daresbury, WarringtonWA4 4AD, U.K.

## Abstract

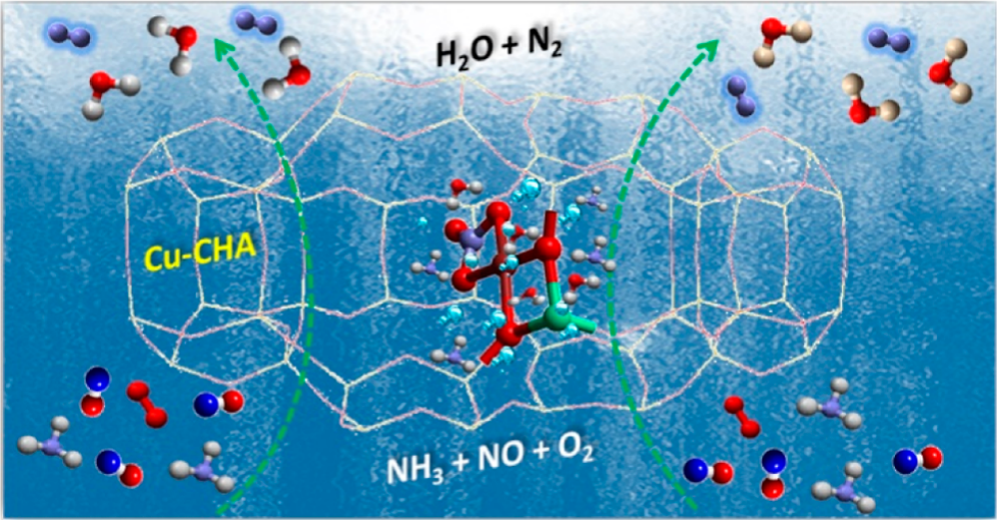

The copper-exchanged zeolite Cu-CHA has received considerable attention in recent years, owing to its application in the selective catalytic reduction (SCR) of NO_*x*_ species. Here, we study the NH_3_-SCR reaction mechanism on Cu-CHA using the hybrid quantum mechanical/molecular mechanical (QM/MM) technique and investigate the effects of solvent on the reactivity of active Cu species. To this end, a comparison is made between water- and ammonia-solvated and bare Cu species. The results show the promoting effect of solvent on the oxidation component of the NH_3_-SCR cycle since the formation of important nitrate species is found to be energetically more favorable on the solvated Cu sites than in the absence of solvent molecules. Conversely, both solvent molecules are predicted to inhibit the reduction component of the NH_3_-SCR cycle. Diffuse reflectance infrared fourier-transform spectroscopy (DRIFTS) experiments exploiting (concentration) modulation excitation spectroscopy (MES) and phase-sensitive detection (PSD) identified spectroscopic signatures of Cu-nitrate and Cu-nitrosamine (H_2_NNO), important species which had not been previously observed experimentally. This is further supported by the QM/MM-calculated harmonic vibrational analysis. Additional insights are provided into the reactivity of solvated active sites and the formation of key intermediates including their formation energies and vibrational spectroscopic signatures, allowing the development of a detailed understanding of the reaction mechanism. We demonstrate the role of solvated active sites and their influence on the energetics of important species that must be explicitly considered for an accurate understanding of NH_3_-SCR kinetics.

## 1 Introduction

Atmospheric NOx emissions, consisting mainly of NO, NO_2_, and N_2_O, are highly damaging to the environment, producing smog and acid rain; they are also “greenhouse gases” and pose a serious hazard to human health both through direct exposure and *via* the formation of ozone.^1,2^ Human activity has had a dramatic impact on NOx levels through fossil fuel combustion, which releases both NO and NO_2_ into the atmosphere.^3^ To mitigate this problem, transition metal catalysis can be harnessed to remove NOx efficiently from exhaust gases through the selective catalytic reduction (SCR) reaction, in which ammonia is used to reduce these species to nitrogen.^4,5^ The major exhaust gases from diesel engines are NO (>90%) rather than NO_2_; therefore, the reduction of NOx occurs mostly *via* what is known as the “standard SCR” reaction.^6^

Small-pore zeolites containing copper such as Cu-SSZ-13, possessing the chabazite (CHA) topology, have shown outstanding performance in the NH_3_-SCR reaction.^2,7,8^ It has been reported that Cu-CHA has not only several active sites, including Cu^+^ and Cu^2+^ ions in NH_3_-SCR, but also species beyond single ions and with higher Cu-ion nuclearity.^9,10^ For low-temperature SCR (<200–250 °C), it has been proposed that Cu ions of higher nuclearity are active, which is, however, not the case for high-temperature SCR where ions of low nuclearity have been proposed.^11^ In the NH_3_-SCR cycle, the reaction follows either a standard SCR (eq 1) or NO-activation pathway (eq 2), which can proceed with the same reduction step as the fast-SCR (eq 3); however, in the “NO-activation” cycle, the rate does not depend on the concentration of NO_2_, while it is dependent on the concentration of NO_2_ in the fast SCR.^12^
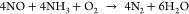
1
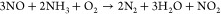
2
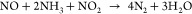
3

Water and ammonia vapors are among the key components of the NOx-containing exhaust gases, and their inevitable presence could lead to adsorption on the transition metal sites, affecting the energetics of the intermediate species.^13,14^ Liu *et al.*(15) investigated the effect of water on NH_3_-SCR activity over Cu-LTA and found a promoting effect of water on low-temperature SCR activity with a plausible solvated [H_2_O–Cu–NH_3_]^+^ species. Similarly, the experimental study by Yu *et al.*(16) over Cu-SAPO-34 also showed a promoting effect of water and reported that in its presence, the reducibility of Cu^2+^ species at high temperature is improved, while NH_3_ oxidation is inhibited. It is also found that ammonia does not block the formation of nitrates when water is present in the feed, as reported by Lee *et al.*(17) Moreover, some studies show that enhanced Cu-ion mobility is caused by ligating water and ammonia molecules, leading to better low-temperature NH_3_-SCR activity.^10,15,18^

To optimize the catalytic process, not only the chemistry of active sites and the intrinsic NH_3_–NO reaction kinetics need careful attention but also the diffusion of the counterion must be understood, which is strongly affected by the ligation of solvent molecules such as H_2_O and NH_3_. Generally, Al sites are responsible for restricting the mobility of counterions, due to electrostatic attraction to the framework. [Cu(NH_3_)_2_]^+^ mobility inside the CHA framework at the sub-second time scale is predicted by Paolucci *et al.*(10) who found a displacement of 9 Å for [Cu(NH_3_)_2_]^+^ by *ab initio* metadynamics. Furthermore, employing molecular dynamics (MD) simulation, O’Malley *et al.* found that the strong coordination of NH_3_ with Cu^2+^ in the center of the CHA cage hinders the interaction of other molecules with the Cu sites.^19^

**Figure 1:**
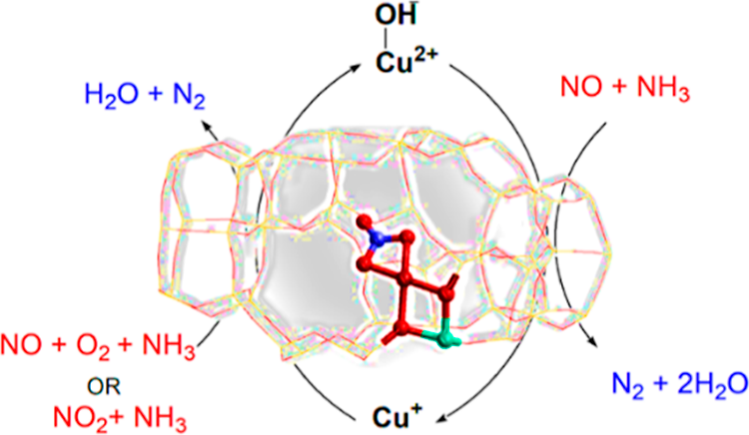
Schematic representation of the catalytic cycle of NH_3_-SCR of NO_*x*_.

It is possible to split the NH_3_-SCR redox cycle and separate the oxidation from the reduction step, which is achieved experimentally by switching between NH_3_ + NO and NO + O_2_^19^ atmospheres to study the individual half-cycles.^12^ The Cu(II)-nitrate species formed as a result of the NO + O_2_ oxidation process can be converted back to the Cu(I) state under NH_3_ + NO reductive conditions (Figure 1). Furthermore, NO can react with the nitrate intermediate to generate gaseous NO_2_ and Cu-nitrite species. Such NO activation, which has been reported for both single Cu-sites and Cu(II)-pair-mediated systems, accounts for Cu(II) reduction and is often considered necessary for the NH_3_-SCR reaction.^5,20^ The formation of NO_2_ species facilitates the oxidation half-cycle and hence leads to establishing a link to the fast SCR reaction.^21^

To understand how solvated Cu cations can control nitrogen chemistry in CHA zeolite, we investigate reaction mechanisms with both modeling and experimental techniques. For this purpose, we have employed density functional theory (DFT) using a quantum mechanical/molecular mechanical (QM/MM) methodology, as implemented in the ChemShell software, and DRIFTS which allow us to observe the formation and consumption of short-lived intermediates in the catalytic reaction. Hereby, we elucidate the influence of physisorbed solvents on the reactivity of Cu-CHA sites and their effect on the energetics of intermediates. We report a comprehensive study in which the computational analysis provides a clear assignment of all main spectroscopic features of the NH_3_-SCR catalytic cycle, which are in good agreement with experiment. Thus, our investigation provides new insights into the NH_3_-SCR reaction by understanding the chemistry of the solvated-Cu-CHA sites and their impact on the key steps in the reaction.

## 2 Methods

### 2.1 QM/MM Methodologies

A hybrid QM/MM approach^22,23^ using the ChemShell software^24−26^ was employed to study the NH_3_-SCR process over Cu-CHA. The model involves a quantum mechanical description of a relatively small cluster of ∼200 atoms around the reaction site, embedded in a much larger cluster modeled using a classical molecular mechanics forcefield. This approach is well suited for describing a local active site, as an alternative periodic DFT approach would require a large, supercell calculation to avoid spurious interactions between the periodically repeated reaction sites. It is also more straightforward in a non-periodic model to increase the accuracy of the QM description through the use of hybrid DFT functionals, as in the current study. Furthermore, the description of the electrostatic environment is more efficiently handled in the QM/MM model through a combination of MM atoms and point charges as detailed below.

**Figure 2:**
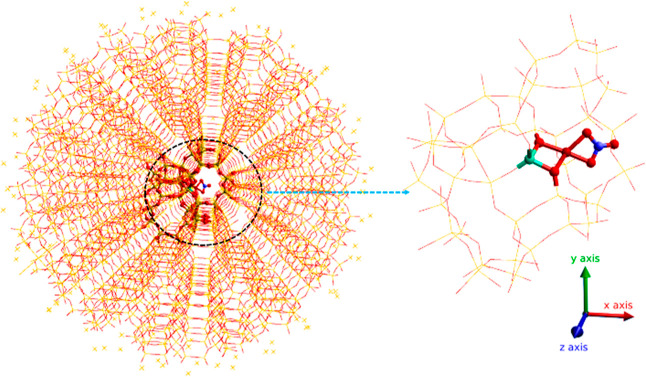
QM/MM embedding setup; CHA cluster (left) with a quantum mechanical region containing nitrate species (right). The outermost region contains point charges to ensure that the Madelung potential in the center of the cluster is accurately reproduced. Atom color codes: Cu (brown), Al (green), Si (yellow), O (red), N (blue), and H (white).

In our CHA-cluster model, we set an active region, where all constituent atoms are allowed to relax freely, extending to a radius of 15 Å (∼28.3 Bohr) from the chosen center containing the Al site. A central core of 143 framework atoms and a charge-balancing Cu cation (not including hydrogen link atoms) is described at the QM level (Figure 2). The active region is in turn surrounded by a frozen spherical layer with a thickness of *ca.* 15 Å. The total number of atoms in the model is 6007, of which 700 are active. A judicious choice of the basis set discussed further below allows us to employ moderate basis sets in the QM region; for example, in the nitrate case, there are 2983 and 2797 Cartesian and harmonic basis functions, respectively, for the largest systems of interest (containing intermediate reacting species). The calculations for the QM clusters were performed using the GAMESS-UK package,^27^ while for the MM part, the DL_POLY package^28^ was employed, with the Hill-Sauer molecular mechanical forcefield,^29^ which assumes that the atoms bind to each other by polar covalent bonds. Further we introduced the outer shell of point charges whose values have been fitted to reproduce accurately the electrostatic field in the active region of the infinite CHA-zeolite framework.

Atoms at the interface between QM and MM regions are connected by bonds that need careful treatment including terminating the QM region by adding hydrogen atoms, forming O-H groups, and use of modified MM charges at the boundary.^23,26^ We divided the QM region into two parts and employed a dual basis set strategy: the number of atoms in the innermost QM1 region is ∼28 including the intermediates which are treated with the triplet-ζ basis set def2-TZVP,^30^ while the outer QM2 region (115 atoms), which includes the terminating link H atoms, is treated with a smaller split valence with polarization def2-SVP^30^ basis set. To build the QM/MM model, it is necessary to remove the classical charges from the QM centers and make sure that the total charge removed from the system is the same as the total charge of the QM region. The guest species of interest in the zeolite and reactant gaseous species were also treated at the QM level using the higher quality def2-TZVP basis set. The coordinates of all atoms in the QM region and active MM region were fully optimized. To find the most favorable location of Cu^2+^–OH^–^ in Cu-CHA, we performed DFT calculations using a range of exchange–correlation density functionals, as presented in the Supporting Information section, and have selected the B97-2^31^ results as providing the most accurate reaction energies and molecular structures. Therefore, the B97-2 data will be used in the main body of this paper. In particular, we have found that the extraframework Cu ions are more stable in eight-membered rings (8-MR) than 6-MR, by 0.3 eV (*cf*. a similar finding in ref (32)). Considering that adsorbed molecules can diffuse through the larger channels, all the adsorbed species including the intermediates were placed within (or near) the 8-MR of the CHA cage. The water (O-end) and ammonia (N-end) molecules were placed at a distance of 2 Å from the Cu center with the proton pointing away from the adjacent oxygens and intermediate species to avoid artificial trapping in hydrogen-bonding interactions. We note that hydrogen bonding with framework oxygen can influence chemistry at metal sites by affecting the binding of functionally important Cu–H_2_O/NH_3_ units.

For vibrational frequencies at a local active site modeled by the hybrid QM/MM approach, only nuclear displacements of the active sites are included, that is, a frozen phonon approximation from the expanded environment accounting for electrostatic interactions is applied throughout the vibrational calculations. To this aim, we chose an active region that contains atoms around the Al center within three coordination spheres (5T-site), including the atoms of the intermediate species, and calculated normal and localized modes. Full details of the approach used for vibrational frequency calculations are given in the Supporting Information section.

### 2.2 Choice of the Model Cluster

To perform the QM/MM calculation, we first created a spherical embedded-cluster model (Figure 2) of CHA from the unit cell of siliceous CHA^33^ optimized at the MM level using the GULP package.^34^ After creating a simple CHA-cluster model, we constructed active sites. For example, in the case of a Brønsted acid site, we replaced one Si with Al and added a charge compensating proton on a neighboring oxygen atom at a site where it is most accessible to facilitate the reaction. The QM region which is contained within the active part of the model includes atoms from the third oxygen shell from the central T-site; as noted, we have added the hydrogen (link atoms) to saturate the terminal oxygen atoms. Furthermore, we modified the same cluster by incorporating copper into the cage of the CHA framework.

We first optimized the purely siliceous CHA cluster using ChemShell and employed the hybrid QM/MM model for zeolites developed by Sherwood.^35^ The geometrical parameters obtained for the Si-tetrahedral sites by ChemShell employing two representative DFT functionals, B97-2^31^ and BB1K,^36^ were found to be in an accordance with the experimental data (details are in Supporting Information, Tables S1 and S2).

### 2.3 Catalyst Preparation

Synthesis of SSZ-13 zeolite (Si/Al = 13) was performed as reported earlier following the hydrothermal approach.^37^ The *N*,*N*,*N* trimethyladamantammonium hydroxide was used as a structure-directing agent under fluoride media. The protonated zeolite is obtained by calcining the sample in the air, first for 2.5 h at 1 °C min^–1^ to 120 °C and then for 10 h at 4 °C min^–1^ to 550 °C. By employing the wet ion-exchange methodology, a typical amount of H-SSZ-13 is then mixed with a copper sulfate solution (50 mL of a 0.1 M solution of CuSO_4_ per gram of zeolite) under constant heating (80 °C for 2 h) and stirring. The resultant product consists of a well-defined crystal of rhombohedral morphology (2.92 wt % Cu loading) which was washed with water and kept at 80 °C overnight.^38^ To perform operando spectroscopic analysis, the pellets of zeolite (8 mm bore, 1.5-tonne pressure) were prepared which were then crushed and sieved to retain a 250–450 μm fraction for experiments.

### 2.4 Catalyst Characterization

Powder X-ray Diffraction (PXRD) patterns were collected to confirm phase purity and crystallinity on a Rigaku Miniflex diffractometer (Cu Kα_1_, 1.54056 Å), and samples were loaded onto a flat Teflon sample holder. Diffraction patterns were collected between 5.0 and 50.0° in 0.02° steps. PXRD shows that a highly crystalline pure phase of Cu-SSZ-13 is present after calcination, subsequent ion-exchange, and calcination steps (see Figure S6). Energy-dispersive X-ray (EDX) analysis of Cu-SSZ-13 (see Table S19) shows that Cu-SSZ-13 has a composition of 2.92 wt % Cu with a Si/Al = 13 which represents 75% Cu ions exchanged into available H^+^ sites. The Brunauer–Emmett–Teller (BET) method was used in the analysis of the total surface area, and the *t*-plot method was used in the micropore volume (Table S20).

### 2.5 DRIFTS Operando ME Experiments

DRIFTS spectra were recorded on a Bruker Vertex 70 spectrometer equipped with a liquid-N_2_ cooled HgCdTe detector and a Praying Mantis mirror unit (Harrick). The spectroscopic cell connected to heated gas supply lines was equipped with a flat CaF_2_ window (2 mm thick; diameter 25 mm). The outlet of the cell was coupled to a Fourier Transform Infrared (FTIR) spectrometer equipped with a 70 mm path length gas cell heated to 150 °C (Bruker Alpha). The sample was placed in the sample cup of the cell (*ca*. 30 mg, 57 mm^3^) after being dried *in situ* in 10 vol % O_2_/N_2_ (100 mL min^–1^) at 400 °C for 2 h. DRIFTS spectra were collected by accumulating 10 interferograms under 80 kHz scanner velocity (0.9 s per spectrum) and at 4 cm^–1^ resolution. Solenoid valves were used to repeatedly switch between gases during a concentration modulation excitation experiment which was functioned using OPUS software (Bruker).

## 3 Results and Discussion

An NH_3_-SCR reaction mechanism has been proposed by Janssens *et al.*, who show both standard and fast NH_3_-SCR of NOx in a complete cycle that can produce the correct stoichiometry for the reaction.^32^ In contrast, in our study, the focus is on solvent effects on the reactivity of the Cu-CHA, and, as argued, it provides key insights into the reaction mechanism from accurate hybrid-QM/MM calculations and concentration modulation ME DRIFTS experiments. Hybrid-QM/MM investigations, DRIFTS experiment, and in-depth scrutiny of solvent effects allowed us to explore especially the intermediate–solvent interactions and identify the important species participating in the NH_3_-SCR event both on the bare and on the solvated sites. The computational mechanistic study focuses on these results, which complement and give insight into the experimental findings.

### 3.1 Vibrational Study

**Figure 3:**
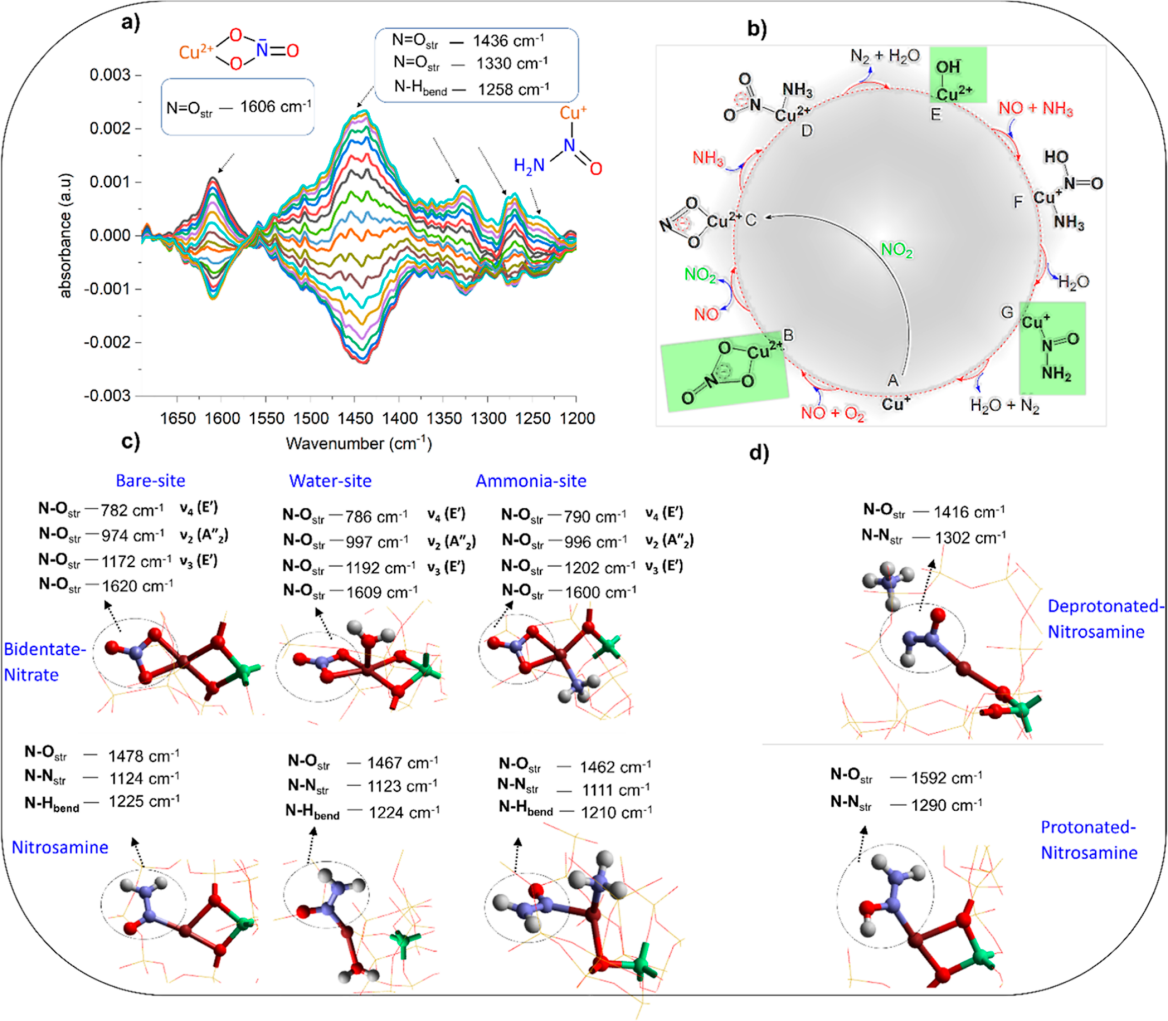
Spectroscopic signatures: (a) concentration modulation ME DRIFTS experiment with the corresponding phase-resolved spectrum, (b) reaction cycle highlighting the identified species, (c) data obtained from QM/MM calculations for bidentate Cu nitrate (Cu–NO_3_) and Cu-nitrosamine (Cu–N(=O)–NH_2_) species for the neutral system, and (d) for the deprotonated and protonated system. Color codes: Cu (brown), Al (green), O (red), N (blue), and H (white). The framework SiO_2_ is shown using a wire framework motif.

First, we performed DRIFTS experiments exploiting (concentration) modulation excitation spectroscopy (MES) and phase-sensitive detection (PSD) to determine the evolution of species in response to a stimulus; in this case, the NO flow during a constant stream of 500 ppm NH_3_ and 10,000 ppm O_2_ produces N_2_ akin to the standard NH_3_-SCR reaction at 250 °C.^38^ This approach allows us to observe the formation and consumption of short-lived intermediates in the catalytic reaction, crucially, the detection of species, which had not been previously observed experimentally. The data are shown in Figure 3a.^38^ Notable observations included the initial consumption of [Cu^2+^(OH)]^−^ to form an important intermediate, which has been identified as copper nitrosamine (Cu–N(=O)–NH_2_) based on the observation of evolving bands in the IR spectrum at 1436 cm^–1^ (N=O_str_), 1330 cm^–1^ (N=O_str_), and 1258 cm^–1^ (N–H_bend_). Below 1200 cm^–1^, we are unable to collect reliable spectroscopic data due to the overlapping of zeolite framework vibrations with other bands. The next species detected in the cycle was a bidentate nitrate (N=O_str_ at 1606 cm^–1^). Note that all bands appear with a different “phase”, or time during the experiment, indicating that the corresponding species are not typically present at the same time. Furthermore, Figure S5 shows the 2400 DRIFTS spectrum collected throughout the course of the modulation experiment in which NO is turned “on/off” repeatedly between 0 and 500 ppm, while the concentration of other reactive components, NH_3_ and O_2_, remains constant. Note that no discernible changes can be detected since the continuous presence of NH_3_ and products of the reaction (H_2_O) dominate the spectrum, particularly in the region between 2500 and 3500 cm^–1^.

Second, we calculated the harmonic vibrational spectra of selected intermediates. The harmonic values obtained were scaled using vibrational scaling factors (see Supporting Information), which were determined by comparing experimental and computational harmonic values using a representative set of gas-phase molecules. We focused on bands of nitrosamine (N–O_str_ and N–N_str_) and nitrate (N–O_str_); the yielded scaling factor to calculate the vibrational frequency of N–O_str_ of nitrosamine is 0.915, while for the N–N_str_, it is 0.918. Similarly, the corresponding scaling factor we applied for the N–O_str_ of nitrate is 0.943.

As shown in Figure 3b, for the bare Cu-nitrosamine (Cu–N(=O)–NH_2_) (species G, Figure 3b) species, we found a vibrational stretching band around 1478 cm^–1^ compared to our experimental value of 1436 cm^–1^, tentatively ascribed to the N=O_str_ mode. Haszeldine and Jander reported the N=O_str_ band at 1488 cm^–1^ which is close to our calculated frequency.^39^ Also, bands in the region 1408–1486 cm^–1^ have been reported by Piskorz and Urbanski^40^ and Tarte^41^ and assigned to the N=O_str_ frequency of nonassociated dialkylnitrosamine (in nonpolar solvent: CCl_4_). They also reported peaks for N=O_str_ between 1346–1265 and 1321–1292 cm^–1^, which can be broad and strong or of medium strength. Importantly, this can be also seen in our measured DRIFTS spectra [(N=O_str_), 1330 cm^–1^]. Furthermore, Kedrova *et al.* have observed the vibrational frequencies of associated nitrosamine and identified both N=O_str_ (1470–1495 cm^–1^) and N–N_str_ (1055–1060 cm^–1^) bands.^42^ On physisorption of water and ammonia at Cu sites, we observed the N-O_str_ band shift down to 1467 and 1462 cm^–1^, respectively. The lower frequency spectral features found in our QM/MM calculations are, in particular, due to N–N stretching vibrations of Cu-nitrosamine (Cu–N(=O)–NH_2_) species. For the bare site, the N–N stretching frequency is calculated as 1124 cm^–1^ as compared to the reported experimental values in the nitrosodimethylamine (1052 cm^–1^) and di-*N*-nitroso-pentamethylenetetramine (1106 cm^–1^).^40^ For the water- and ammonia-solvated sites, the N–N_str_ is found at 1123 and 1111 cm^–1^, respectively. In addition to the N=O_str_ and N–N_str_ bands for nitrosamine species, we also examined the N–H bend, which is likely to appear in this region; the computed value for the bare site is at 1225 cm^–1^, whereas for the water- and ammonia-solvated sites, it is found at 1224 and 1210 cm^–1^, respectively. The reduction of the vibrational frequency of these modes shows the ligand effect as stronger ligands may weaken the bonding.

Next, we investigated a bidentate nitrate species (species B, Figure 3b) with a focus on the N=O_str_ stretching mode. Generally, nitrate species have four notable modes.^43,44^ Our calculations show that the bare bidentate Cu–NO_3_ structure possesses the main N=O_str_ stretching frequency of 1620 cm^–1^, while for the physisorbed water and ammonia, it is found at 1609 and 1600 cm^–1^, respectively. All these three bands are quite close to our experimental DRIFTS value of 1606 cm^–1^ and to earlier reports^45,46^ of the nitrate N=O_str_ difference FTIR band in Cu-SSZ-13. In addition, small broad bands that appeared in our experimental findings in the region of 1230–1250 cm^–1^ could be assigned to the antisymmetric stretch (ν_3_ (E′)) of the N–O band, as reported by Zapata and García-Ruiz.^43^ However, our QM/MM calculation shows this band at relatively lower frequencies for this mode; for instance, it is seen at 1172 cm^–1^ for the bare site, while for physisorbed water and ammonia sites, it appears at 1192 cm^–1^ and 1202 cm^–1^, respectively. Finally, two bands that originated by out-of-plane and in-plane deformation modes for nitrate species are displayed. The out-of-plane deformation band (ν_2_ (A_2_″)) is normally located within the range of 800–900 cm^–1^, while the in-plane band (ν_4_ (E′)) ranges from 700 to 780 cm^–1^ in nitrate salts.^43,44^ Our calculated out-of-plane deformation occurs at a somewhat higher frequency, as seen in Figure 3b; however, the in-plane deformation has appeared almost in the same region as previously reported.^43^

One of the significant effects of solvation is the possibility of site deprotonation due to a proton transfer to solvents even if only as a transient species. Therefore, we completed this analysis by studying vibrations of a negatively charged intermediate species that would result from such deprotonation by possibly abstracting the proton from the −NH_2_ group of nitrosamine to form NH_4_^+^ ions (see Figure 3d). The calculated N–O_str_ (1416 cm^–1^) band is found at somewhat lower frequencies for the deprotonated nitrosamine NH_4_^+^(Cu–N(=O)–NH) species compared to the neutral system (1478 cm^–1^), while the N–N_str_ stretching bands for this species appear at a relatively higher frequency of 1308 cm^–1^ than the neutral system (1124 cm^–1^). There is a mixed asymmetric stretch mode of the NH_4_^+^ group combined with N–N_str_, which may be the reason that N–N_str_ appears at a higher frequency than expected.

Inversely to the deprotonation of nitrosamine species, we also examined the likely protonation of Cu-nitrosamine (Cu–N(=O)–NH_2_) that generates an −OH site, that is, (Cu–N(=OH)–NH_2_). The calculated N–O_str_ band (1592 cm^–1^) in this case was found at significantly higher frequencies compared to both deprotonated nitrosamine (1416 cm^–1^) and neutral system (1478 cm^–1^). Based on the result obtained, we infer that the experimental DRIFTS data show either neutral or deprotonated nitrosamine rather than protonated species. Moreover, we also carried out a separate vibrational analysis on Cu–(N(=O)–OH)–NH_3_ (species F, Figure 3b), a postulated species which further decomposes to the (Cu–N(=O)–NH_2_) species. The N–O_str_ band appeared for this intermediate at 1585 cm^–1^ (Figure S2).

Furthermore, we examine the N=O_str_ vibrational mode of bidentate-nitrite (Cu–NO_2_) (species C, Figures 3b, S3) to determine whether the 1606 cm^–1^ band in the DRIFTS spectrum originates from the nitrate or nitrite. The computed N=O_str_ band of this particular species is, however, found to be significantly lower than the N=O_str_ band of the bidentate-nitrate species at 1273 cm^–1^, while for the solvated H_2_O and NH_3_ active sites, it is observed at 1263 and 1260 cm^–1^, respectively. Notably, the calculated bands are comparable with the experimental values of *ca.* 1229 cm^–1^ for the isostructural linear nitrite species reported in refs (47) and (48).

We have performed a similar calculation on (Cu–(NO_2_)–NH_3_) (species D, Figures 3b and S4); however, again we found that the NO band for this species is lower than the 1606 cm^–1^ band in the DRIFTS spectrum assigned to N=O_str_ vibration for all three sites. Based on the calculated vibrational modes, we infer that the spectroscopic signatures that appear in the DRIFTS spectrum can be tentatively assigned to the nitrate and nitrosamine species. Furthermore, we do not detect the N–O signatures in the DRIFTS spectrum for the nitrite (species C) and the species D and F in the cycle and even the first Cu–NO(OH) interactions on NO adsorption, indicating that these species are too short-lived to be observed experimentally. Hence, we propose the assignment of the DRIFTS spectral features summarized in Table 1 and shown in Figure 3a.

**Table 1:** Features Observed in a Concentration ME DRIFTS Experiment’s Time-Resolved Spectrum under SCR Conditions (NO Gas Switch Pulse Sequence)^a^

DRIFTS (cm^–1^)	vibrational mode	strength	width	calculated IR mode^b^	ref
1606	N=O stretch (nitrate)	medium	sharp	1600 (B), 1609 (H), 1620 (N)	(45, 46)
1436	N=O stretch (nitrosamine)	strong	sharp	1478 (B), 1467 (H), 1462 (N)	(39−42)
1330	N=O stretch (nitrosamine)	small	medium		(41)
1258	N–H bend (nitrosamine)	small	sharp	1225 (B), 1224 (H), 1210 (N)	
1230–1250	N–O*anti*-symm. stretch (nitrate)	small	broad	1172 (B), 1192 (H), 1202 (N)	(43, 44)
1210	N–N stretch	small	medium	1124 (B), 1123 (H), 1111 (N)	(40, 42)

a Assignment is based on an analysis of the literature and calculated vibrational spectra of reactant intermediates shown in Figure 3b.

b B, bare; H, physisorbed H_2_O; and N, physisorbed NH_3_.

Furthermore, we examine the O–H and N–H_str_ vibrational features for the competing [Cu^2+^(OH)]^+^ and Brønsted acid sites under an NH_3_-SCR environment. In the DRIFTS spectrum (Figure S5), there are multiple vibrational bands in the region between 2500 and 3500 cm^–1^ as the spectrum is mostly dominated by NH_3_ and H_2_O. The most notable feature in the phase-resolved spectrum is, however, that at 3655 cm^–1^, which is indicative of [Cu^2+^(OH)]^+^ species that exhibit a greater fluctuation than those related to silanol groups or bridging hydroxyls, which is also reported by Giordanino *et al.*(49) Our calculated value (3668 cm^–1^) is in good accordance with the DRIFTS experimental value and with the reported data.^45^ The experimental attribution of this band to a [Cu^2+^(OH)]^+^ species was based on its response to changes in gas composition, indicating that this species actively participates in the catalytic mechanism.^38^ The vibrational signatures of the likely NH_3_ adsorption on the Cu^2+^ active sites are also confirmed by theory and experiment. In the corresponding DRIFTS spectrum, the N–H band which appears at 3332 cm^–1^ is in good accordance with the computed value of 3336 cm^–1^. Moreover, we detect the N–H bending features at 1620 cm^–1^ in the DRIFTS spectrum which is reproduced in our calculations with an accuracy better than 1 cm^–1^ and agrees well with previous work.^50^ To study the competitive reaction pathway between NH_3_ adsorbed on Cu sites and Brønsted sites, we analyzed the vibrational signatures of the NH_3_ adsorption on the Brønsted acid site, the interaction which may lead to evolution of NH_4_^+^ ions owing to the NH_3_ protonation over these acid sites. The band intensity grows from *ca.* 1454 cm^–1^ in the DRIFTS spectrum (see Figure S5), indicating the consumption of the *v*(O–H) band associated with the Brønsted acid sites. The position of this band is confirmed by our computed value of 1455 cm^–1^ and previous work^49^ for NH_4_^+^ ions. The N–H stretching band of NH_4_^+^ ions appears at 3272 cm^–1^ in the DRIFTS spectrum, which also agrees with the calculated vibrational value at 3278 cm^–1^.

Our computational analysis provides a clear assignment (Table 2) of all main spectroscopic features of the species presented in Figure 3b.

**Table 2:** Calculated Vibrational Bands of Key Intermediate Species Presented in the NH_3_-SCR Catalytic Cycle—See Figure 3b

species	wavelength (cm^–1^)^a^	description of IR active mode
Cu–NO_3_ (species B)	1600 (B), 1609 (H), 1620 (N)	N=O stretch
Cu–NO_2_ (species C)	1273 (B), 1263 (H), 1260 (N)	N=O stretch
(Cu–(NO_2_)–NH_3_) (species D)	1501 (B), 1481 (H), 1464 (N)	N=O stretch
	3445 (B), 3450 (H), 3447 (N)	N–H stretch
Cu–(N(=O)–OH)–NH_3_ (species F)	1585 (B), 1579 (H), 1575 (N)	N=O stretch
	3313 (B), 3471 (H), 3485 (N)	N–H stretch
	3197 (B), 3216 (H), 3226 (N)	O–H stretch
(Cu–N(=O)–NH_2_) (species G)	1478 (B), 1467 (H), 1462 (N)	N=O stretch
	3482 (B), 3493 (H), 3517 (N)	N–H stretch
[Cu^2+^(OH)]^+^ adsorbed NH_3_	3336	N–H stretch
	3668	O–H stretch
	1620	N–H bend
Brønsted acid site adsorbed NH_3_	3279	N–H stretch
	1455	N–H bend

a B, bare; H, physisorbed H_2_O; and N, physisorbed NH_3_.

### 3.2 Comparison of Reaction Energetics with Experiment

Next, we assess our computational approach for the overall de-NOx reaction energy that involves NO activation to stable long-lived intermediates and products. To this end, we compare the simulated data with experimental measurements and analyze the results obtained (Tables S3 and S4). First, we calculated the reaction energy for eq 2, an NO-activation pathway using computational parameters described in Section 2.1 (B97-2^31^ functional and the def2-TZVP^30^ basis set). The overall experimental Δ_*f*_*H*_0_° value is −9.01 eV (−869 kJ/mol), which is calculated from the standard enthalpy of formation (Table S5) of the gaseous species involved in this reaction. The calculated theoretical value is −8.37 eV, which is similar to the previous calculations (∼−8.45 eV, estimated from Figure 3 of ref (32)). Thus although the two theoretical estimates are within 0.08 eV of each other, there remains a small but significant deviation from the experimental value.

### 3.3 Cu Displacement on Solvation

**Figure 4:**
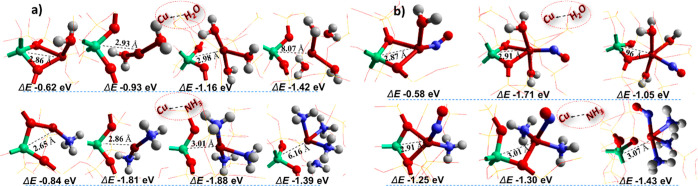
Interaction of physisorbed (a) water and ammonia with Cu(I)-CHA active sites, and (b) behavior of NO on solvated Cu(I)-CHA site. The model used is shown as an extra framework. Color codes: Cu (brown), Al (green), O (red), N (blue), and H (white).

The promoting effect of water on the migration of Cu species suggested that unanchored Cu ions migrate to form active sites which can promote the SCR reaction.^18^ In our calculations summarized in Figure 4a, we observed that an increase in water coordination to the Cu-active sites displaces the Cu species away from the CHA framework. As the number of H_2_O molecules around the Cu(I)-CHA increases, the interaction between the framework and Cu species becomes weaker. In the case of four H_2_O molecules, the distance between Al and Cu is calculated to be 8.07 Å, consistent with the understanding that water molecules promote the mobility of Cu ions. Similarly, the effect of NH_3_ solvation during the oxidation part of the SCR reaction is crucial. Some previous reports suggest that the NH_3_-solvated Cu(I) sites interact weakly with the zeolite framework and move away as a mobile species to react with the O_2_ and yield an O-bridged Cu(II) dimer;^51^ we find an increase in bond length between Al and Cu as the number of NH_3_ molecules increases, suggesting that an enhanced number of NH_3_ molecules can detach Cu species from the framework. *Ab initio* molecular dynamics (AIMD) simulations show that after the adsorption of one NH_3_ molecule in Cu-SAPO-34^52^ at 298 K, the Cu^+^ cation is somewhat displaced yet remains coordinated with one of the framework oxygens in an H_3_N–Cu–O bond, which is also evident from experiments on Cu-SSZ-13.^49^ These studies demonstrate that the Cu–O bond in the Cu^+^–NH_3_ system is broken by the adsorption of a second NH_3_ molecule, resulting in the formation of a linear Cu^+^(NH_3_)_2_ complex that can move readily inside the pore. Our QM/MM simulations agree with these findings, as can be observed from Figure 4a. Furthermore, Paolucci *et al.*(10) used *ab initio* metadynamics and a supercell with a minimum image distance in excess of 10 Å to evaluate the mobility of Cu^I^(NH_3_)_2_ complexes over time scales which are inaccessible to conventional AIMD. Their results supported by experimental observations show a ∼9 Å diffusion length for a Cu ion that penetrates an 8-MR window separating two adjacent CHA cages. In our study, we employed a more detailed model of the solvent behavior in the Cu-SSZ-13 system. Similar to the abovementioned study, we start our analysis with a single-solvent adsorbate molecule simulating both water and ammonia. Next, we introduced stepwise a series of solvent molecules bringing the solvent content to four molecules per cage. Upon adsorption, we observed a pronounced displacement of the Cu ion away from the framework Al site. In the case of water, the calculated distance between Al and Cu is 8.07 Å, while for the solvated Cu–NH_3_ case, it is 6.16 Å, consistent with the understanding that solvent molecules promote the mobility of Cu ions. In contrast to the report by Paolucci *et al.*,^10^ we do not observe the penetration through the 8-MR window, which could be attributed to the different boundary conditions in our simulations. Notably, the Cu ions in our study are not forced to move away from the Al site but displace spontaneously as a result of geometry optimization from their initial position close to the Al site in response to the strong interaction of the polar solvent with both framework and extraframework metal cations.

We also examined the approach of NO toward the solvated Cu(I)-CHA and infer that in the presence of water, the effect of NO is vital, as it restricts, to some extent, the displacement of the Cu-ion species from the framework (Figure 4b). This is apparent from the corresponding separation distances (2.96 Å) between Cu and Al when compared with those when NO is not present, which can prevent Cu(II) dimer formation and promotes the formation of nitrate species.^53,54^ In addition, it also suggests that the presence of NO might prevent the mobility of Cu ions in the presence of water. We, however, observed a complete detachment of Cu ions from the framework when NO interacts with three NH_3_ coordinated sites with a distance of 3.071 Å between Cu and Al (Figure 4b).

### 3.4 Adsorption Study

We examine first the binding energies of the gaseous reactant molecules both on the Cu(I)-CHA and Cu(II)-CHA sites.

**Figure 5:**
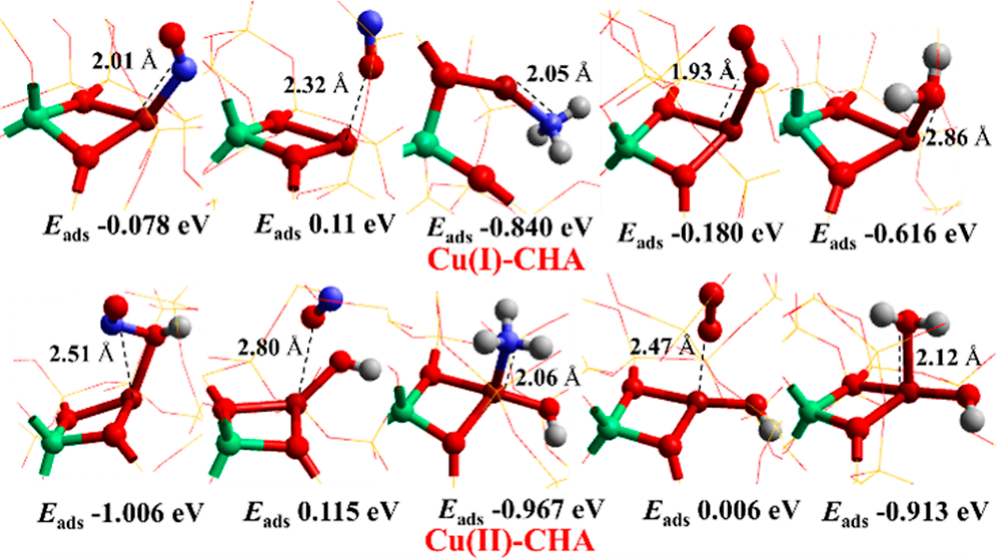
Reaction adsorption energies of NH_3_, NO (with both the O- and N-end down), H_2_O, and O_2_ on Cu(I)-CHA and Cu(II)–OH/CHA sites. The model used is shown as an extra framework. Color codes: Cu (brown), Al (green), O (red), N (blue), and H (white). The framework SiO_2_ is shown using a wire framework motif.

From the results reported in Figure 5, considering the behavior on the Cu(I) site, we note that the adsorption of NO, NH_3_, and H_2_O on the Cu^+^-/CHA site is, as expected, exothermic. The binding energy for NH_3_, yielding NH_3_–Cu^+^, is calculated as −0.84 eV (−81 kJ/mol)^32,55^ where the reported average experimental heat of adsorption for ammonia on Cu-Beta^56^ and Cu-CHA^57^ is nearly −100 kJ/mol. The experimental values, however, should be treated with caution as Cu-exchanged zeolites would still have a significant fraction of strongly adsorbing Brønsted acid sites.^58,59^ We have also examined the interaction of ammonia with the Brønsted acid site and found a binding energy of −1.1 eV (−106 kJ/mol), which is appreciably higher than that of the Cu Lewis site and is close to the experimental report. The heat of NH_3_ adsorption over non-exchanged zeolites H-CHA has been reported to be as high as −145 kJ/mol (obtained using microcalorimetry techniques),^60^ which is appreciably stronger and could be related to the Brønsted acid complexes, which warrants a separate investigation.

Furthermore, the reported studies also suggest that the heat of adsorption decreases with increased ammonia coverage.^57^ It is reported that the binding of NH_3_ on Cu^+^ strongly influences the interaction of Cu with the framework of CHA; however, depending on the number of NH_3_ molecules bound and temperature, cation mobility can become possible.^52^ In this context, we observed that a single physisorbed NH_3_ molecule detaches Cu^+^ from one of the framework O atoms, making a new coordination adduct, H_3_N–Cu–O, with a distance between Al and Cu of 2.645 Å. H_2_O also exothermically binds to the Cu(I)-CHA sites with a binding energy of −0.62 eV (−59 kJ/mol), but the monovalent Cu remains attached to the two framework O atoms. Furthermore, Lercher and co-workers employed a periodic PBE + D3 approach and obtained an adsorption energy of −77 kJ/mol for water on the Brønsted acid site of H-MFI,^61^ whereas a value of −78 kJ/mol is obtained for the H-CHA by a hybrid MP2/PBE calculation.^62^ We also observed that NO weakly adsorbs on bare Cu(I)-/CHA, through the N atom (with a binding energy of only −0.08 eV); however, it is more favorable on solvated ammonia Cu(I) (−1.25 eV) and water Cu(I) (−0.58 eV) sites, where the corresponding experimental heat of adsorption is −65 kJ/mol (−0.70 eV) on Cu-Beta.^56^

Turning now to the Cu(II) sites, NH_3_ and NO interact favorably with divalent Cu (II)–OH sites. NO binds through the O atom (−OH) (−1.01 eV), suggesting that it preferentially binds to the OH site of Cu(II)–OH, generating an HONO species. The formation of the HONO species has been described earlier.^47,63^ In the case of NH_3_, strong bonding to the Cu(II) site is calculated, with a binding energy of −0.97 eV (−93 kJ/mol), and as seen in Figure 5, the attachment of Cu(II) with the framework O is intact. We also demonstrate that water can interact strongly with the divalent Cu site (with a binding energy of −0.91 eV), suggesting that water can affect the reactivity of active sites.

Based on the interaction of molecules with the active sites, we conclude that the adsorption of NO species with the O-end down is uncompetitive (with a positive energy) on both Cu^+^ (0.11 eV) and Cu^2+^ (0.12 eV) sites, suggesting that the N-end down is the only feasible attachment to Cu-CHA sites. We found that O_2_ preferably binds to Cu(I) sites with a binding energy of −0.18 eV (−17 kJ/mol) as reported before, while on the Cu^2+^–OH^–^/CHA site, the adsorption energy is calculated to be positive (0.01 eV),^52^ which rules out the possibility of O_2_ binding to the Cu^2+^–OH^–^/CHA, hence, signifying that O_2_ plays a key role in the reoxidation of the Cu^+^-site.

### 3.5 Catalytic Cycle

**Figure 6:**
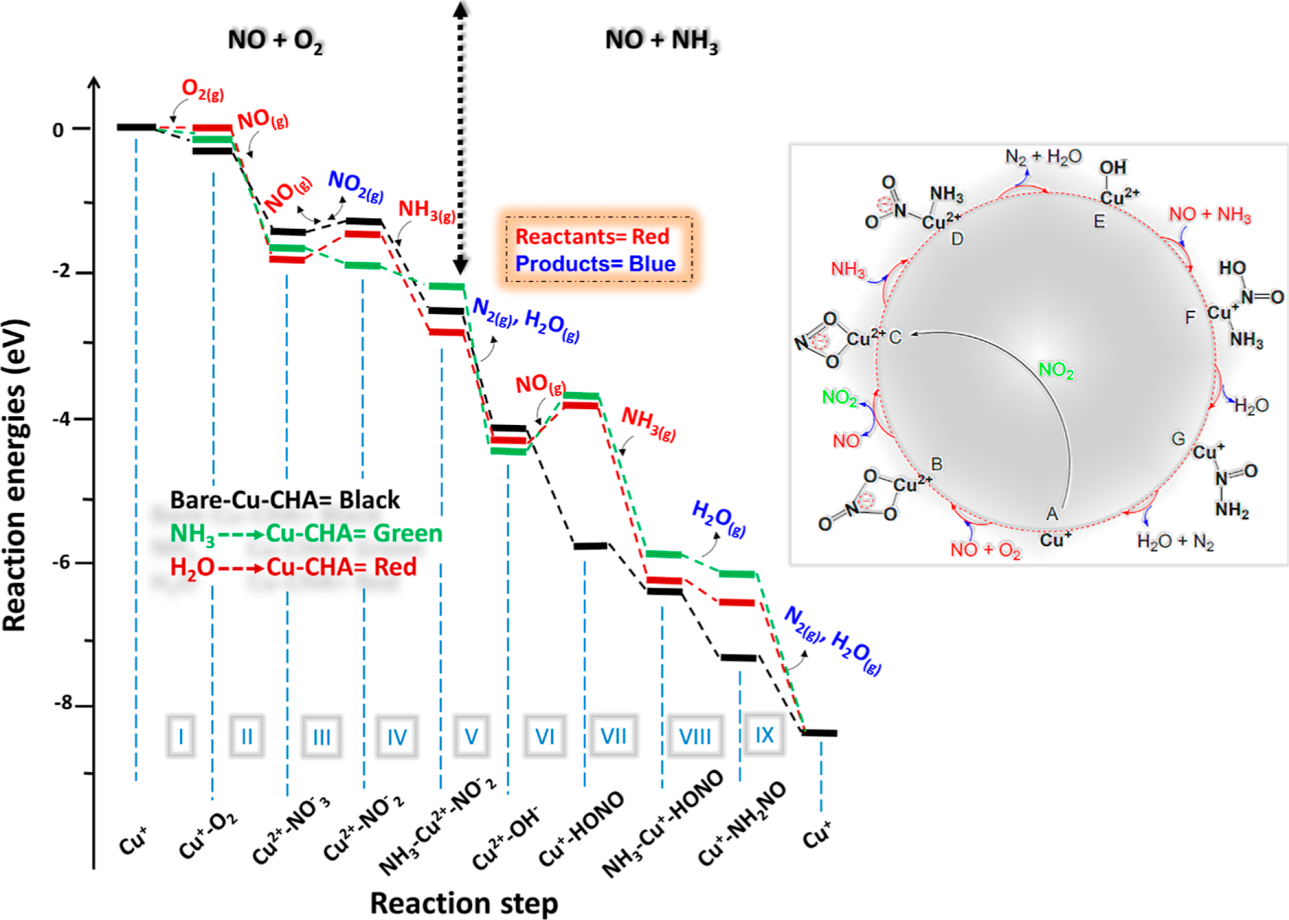
Potential reaction-energy landscape for NH_3_-SCR on the activated Cu-CHA site and (black) on the bare site, (green) with physisorbed ammonia and (red) with physisorbed water. Inset right: NO-activated NH_3_-SCR of the NOx catalytic cycle.

During the NH_3_-SCR cycle, molecules including NO, NH_3,_ O_2_, N_2,_ and H_2_O are either adsorbed or desorbed from the Cu-CHA site, and the intermediate species are generated at each step (Figure 6). The adsorption of NO and O_2_ on Cu^+^-/CHA generates nitrate and nitrite species which undergo decomposition to N_2_ and H_2_O; this half cycle of the NH_3_-SCR is, as noted, known as the oxidation part, which is followed by the reduction of Cu^2+^ where Cu^2+^–OH^–^/CHA reacts with both NO and NH_3_, with Cu^2+^ reduced to Cu^+^ while generating N_2_ and H_2_O as a product, as is evident from both experimental and theoretical studies.^52^ The calculated adsorption energies of the corresponding gaseous species allow us to obtain the reaction energy landscape. Note that in the DRIFTS spectra, all bands appear with a different “phase” or time during the experiment, indicating that the corresponding species are not typically present at the same time and giving further credence to the cyclical nature of the proposed mechanism shown in the inset of Figure 6.

Considering the NH_3_-SCR of the NOx cycle, first, without physisorbed solvent molecules, the corresponding reaction energy landscape is shown in Figures 6 (black lines) and S7. The starting point for the chemical reaction is an isolated Cu(I) site that activates the O_2_ molecule (step I). The energy diagram demonstrates that O_2_ adsorption is exothermic (−0.18 eV) over the Cu(I)-CHA site which is followed by NO adsorption that generates the Cu–NO_3_ species with a formation energy of −1.34 eV while simultaneously oxidizing Cu(I) to Cu(II) (step II). It is evident from previous reports that gaseous NO_2_ can react with the Cu^+^ site, yielding a bidentate Cu nitrite species (step III), which is often considered the fast-SCR reaction.^64^ The reaction of gaseous NO with nitrate^54^ is reported to be a two-step process where first NO coordinates with the nitrate species and then decomposes to nitrite with the release of gaseous NO_2_. To this end, we found a total energy change of +0.17 eV from the nitrate to nitrite step. In the case of the NH_3_ reaction with the nitrite species, an intermediate (step IV) is formed with a formation energy of −1.24 eV. Furthermore, we found that NH_3_ can also react with the Cu–HONO site that generates an NH_3_–Cu–HONO intermediate (step VII) with an H_3_N–Cu bond length of 2.160 Å and a formation energy of −0.63 eV. Subsequently, it decomposes and leads to the generation of an important intermediate, the Cu-nitrosamine (Cu–N(=O)–NH_2_) (step VIII), which is considered to play a key role in the NH_3_-SCR reaction as it involves the formation of the first N–N bond in the N_2_ product. The QM/MM results agree with the sequence of species identified in our DRIFTS data. The existence of both nitrosamine and nitrate in the DRIFTS spectra is also evident from the calculated QM/MM harmonic vibrational data.

#### 3.5.1 Effect of Water Solvation

Water is one of the main products of NH_3_-SCR reaction, and therefore, the hydrated state of the active sites cannot be ignored.^65^ Taking into account the calculated adsorption energies of H_2_O on Cu(I)-CHA (−0.62 eV) and Cu(II)-CHA (−0.91 eV) sites, we suggest that the NH_3_-SCR reaction proceeds differently on solvated Cu-CHA sites. Using the same computational approach, we have studied the effect of water on the formation of the intermediate species; single water molecules are attached to the isolated Cu(I)/Cu(II) active sites to which intermediate species are bound. The corresponding reaction energy landscape is shown in Figures 6 (red lines) and S8. We observe notable differences in the formation energies of some important steps. For example, our results show that without physisorbed water, the formation of nitrate species is less favorable (−1.34 eV) than in the presence of water (−1.69 eV) (step II), suggesting that water can promote the re-oxidation half-cycle, which is also supported by experimental findings as reported by Wan *et al.*,^18^ showing that water could markedly enhance NO reduction. In contrast, we found that on the water-occupied −Cu-CHA site, the formation of Cu-nitrosamine (Cu–N(=O)–NH_2_) (step VIII) is less favorable (−0.11 eV) than in the absence of water (−0.66 eV), suggesting that water can slow down the formation of this species, which can ultimately affect the reduction part of the NH_3_-SCR cycle. We observe that the effect of water on nitrosamine is more significant (+0.55 eV) than on the nitrate (−0.35 eV), which implies that the impact of water could be a (slight) net negative influence on activity. Commensurate with this, it has also been reported previously that residual H_2_O or hydrocarbons can block the active sites or alter their activity.^66^

#### 3.5.2 Effect of Ammonia Solvation

Previous experimental evidence showed that preadsorbed NH_3_ at 300 or 250 °C can enhance NOx reduction; however, low activity of NO reduction was observed below 150 °C.^14^ From the calculated binding energies of NH_3_ for both Cu(I)-CHA (−0.62 eV) and Cu(II)-CHA (−0.91 eV) sites, we can infer that the reactive sites occupied by NH_3_ molecules can influence the mechanism. We have investigated the influence of ammonia on the formation of the intermediate species, as shown in green lines in the energy profile (Figures 6 and S9). Notably, the formation of Cu bidentate nitrate species (Cu–NO_3_) on the physisorbed NH_3_ site is found to be more favorable (−1.59 eV) than with the bare site (−1.34 eV) (step II). In the case of physisorbed water and the bare site, the generation of nitrite under the release of NO_2_ is found to be endothermic, but for physisorbed ammonia, it is exothermic (step III). In addition, the formation of the important intermediate, Cu-nitrosamine (Cu–N(=O)–NH_2_) (step VIII), is less favorable on the physisorbed ammonia active sites (−0.31 eV), when compared to the bare site (−0.66 eV), indicating that physisorbed ammonia can block the active sites for the NH_3_ + NO activation half cycle.

### 3.6 H_2_NNO Decomposition

**Figure 7:**
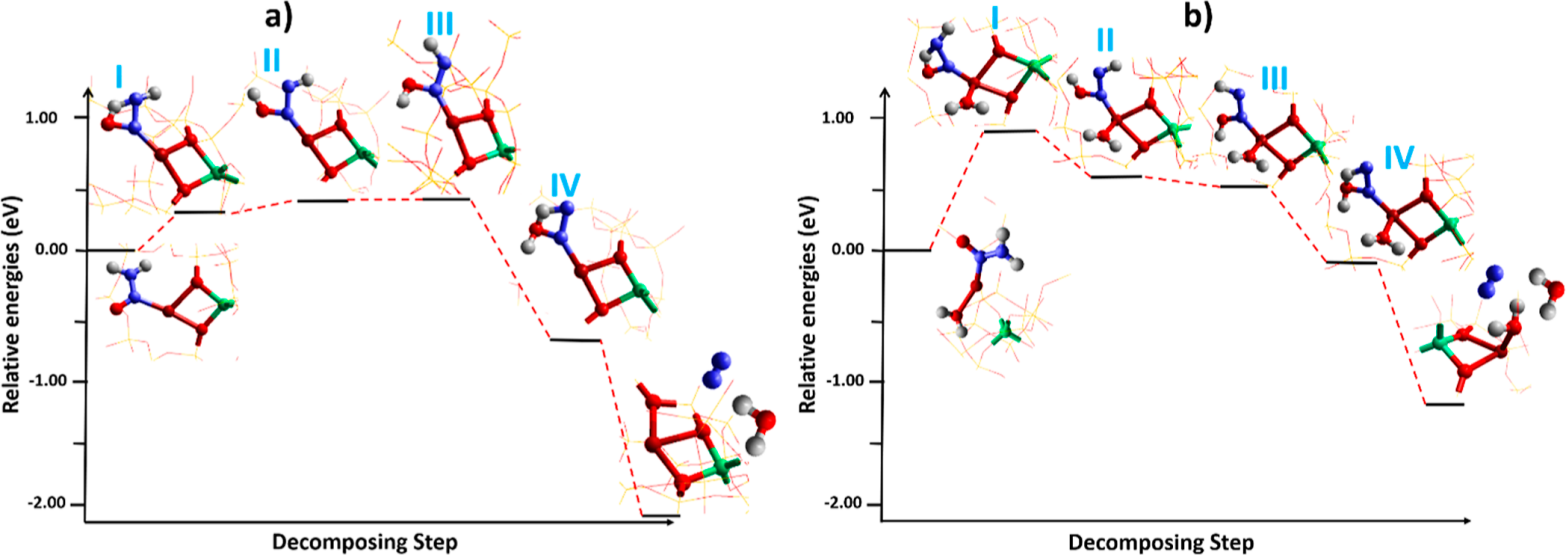
Calculated potential energy surfaces for H_2_NNO isomerization (a) without water and (b) in the presence of physisorbed water on the Cu-CHA-sites. The model used is shown as an extra framework. Color codes: Cu (brown), Al (green), O (red), N (blue), and H (white).

An important step in the NH_3_-SCR mechanism is the reaction of NH_3_ with HONO that generates the NH_3_–Cu–HONO intermediate which eventually leads to the formation of the key intermediate Cu-nitrosamine (Cu–N(=O)–NH_2_), as shown in Figure 6 (step VIII). The decomposition of H_2_NNO has been extensively studied and proceeds through the transformation of several important isomers with high activation barriers.^67−69^ For example, DFT calculations showed that the energy barrier for the H_2_NNO decomposition is considerably lowered *via* proton exchange between the Brønsted acid site and H_2_NNO over V_2_O_5_,^70^ the mechanism of which is similar to dehydrogenation of propane over vanadia.^71^ A similar study has been conducted over Cu-CHA where the decomposition of H_2_NNO is investigated on Brønsted acid sites.^72^ Such a study has also been reported over Cu-SAPO-34^73^ and ZSM-5^74,75^ using a cluster-based computational approach. As it is found that the solvent can coordinate to the Cu-site, we, therefore, investigated the decomposition pathway of H_2_NNO on isolated Cu-CHA active sites both in the presence and absence of physisorbed water (Figure 7). The schematic illustrations of isomeric decompositions of H_2_NNO intermediates are shown in Figure S10. The adduct rearrangement process of H_2_NNO species starts with 1,3 H-transfer, breaking one N–H bond with the transmission of an H to an adjacent O atom, which leads to the formation of an O–H bond. The H-transfer leads to two isomers *cis–trans* (II) and *trans–cis* (III) through the four-membered ring(I). The total energy change from structures (I) to (II) and (III) are calculated as 0.04 and 0.06 eV on the bare site, respectively, while with ligated water, it is −0.33 and −0.46 eV, respectively, suggesting some positive impact of solvated sites on the energetics of this step. From H_2_NNO to HNNOH, the H-transfer is accompanied by shortening of the N–N bond (from 1.35 to 1.299 Å) with lengthening of the N–O bond (from 1.208 to 1.306 Å) that eventually ends with the termination of respective bonds and generation of H_2_O and N_2_ as products. We have noted that the increased coordination of H_2_O molecules detached Cu–H_2_NNO from the framework (Figure S11), showing that the solvent can affect the binding of Cu–H_2_NNO species to the framework.

### 3.7 Reactivity of HONO

We also examined the reactivity of Cu–HONO species for NH_3_, H_2_O, O_2_, and NO, which are the key reactants and products in the NH_3_-SCR reaction. We have found that NO can bind with the Cu(II)–(OH) site forming the Cu–HONO species^20,53,76^ that can react with NH_3_ to yield NH_4_NO_2_, a short-lived and unstable species which can decompose to N_2_ and H_2_O. The subsequent desorption of HONO is endothermic (energy barrier: 0.145 eV) as previously reported;^76^ we, therefore, performed a series of calculations to assess the interaction of other species with the Cu–HONO site, as displayed in Figure S12. Considering the approach of solvent molecules first, we found that the interaction of Cu–HONO with H_2_O is exothermic, while with ammonia, it is endothermic. The binding energy of H_2_O is calculated to be −0.39 eV with a bond distance between O and Cu of 2.155 Å. In the case of NH_3,_ the binding energy is 0.99 eV with a bond distance between N and Cu of 2.160 Å. The interaction of dioxygen with the Cu–HONO site is in the superoxo (O^2–^) mode with an average bond distance of 2.127 Å between O and Cu. We also note the interaction of NO with Cu–HONO, forming a NO–Cu–HONO adduct.

## 4 Summary and Conclusions

The present study has aimed to confirm the identity of key intermediates in the NH_3_-SCR reaction and elucidate the role of physisorbed solvents, as the Cu-CHA active sites will be affected by solvent molecules. The main catalytically active sites that facilitate the adsorption of species are both monovalent- and divalent-copper sites that can drive the NH_3_-SCR to generate important intermediate species including nitrates and nitrites. By understanding the parallels between the water and ammonia interactions with active sites, we find that an increase in solvent coordination to the Cu-active sites liberates the Cu species away from the CHA framework. DRIFTS data showed the formation and consumption of short-lived intermediates in the catalytic reaction, crucially, the detection of important bands for both nitrosamine and bidentate nitrate species, which is in accordance with the calculated frequencies by QM/MM calculation, giving further credence to the proposed mechanism. Our computational analysis provides a clear assignment of all main spectroscopic features of the NH_3_-SCR catalytic cycle, which are in good agreement with experiment. To understand the role of solvents on the kinetics of the NH_3_-SCR cycle, the adsorption of gaseous species and the formation of intermediates and their spectroscopic signatures on the solvated active sites have been investigated which can help tune the rational design of important reaction steps. From the potential energy landscapes, we observed that the formation of nitrate species is energetically more favorable on solvated active sites than on the bare site, suggesting that solvents can promote the re-oxidation part of the NH_3_-SCR cycle. This effect is more significant in the case of water than ammonia. In contrast to the potential benefits seen on the oxidative part, both water and ammonia are found to inhibit the reduction part of SCR since the formation of important intermediates such as Cu-nitrosamine is relatively less favorable on solvated active sites than on the bare sites, suggesting that solvent can slow down the reduction part of the NH_3_-SCR cycle. This finding explains why there is some debate concerning the effect of water and ammonia on the reaction since it seems to affect some parts of the cycle positively and others negatively. In addition, solvating ammonia species were also found to accelerate the oxidation part of the cycle, which is also apparently evident from experimental data showing that preadsorbed NH_3_ at 250–300 °C can enhance the NOx reduction. Our study provides additional understanding, helping to unravel the influence of solvents on the energetics of the active sites, and provides guidance for optimizing the NH_3_-SCR process.

## Supporting Information Available

Additional computational details; benchmarking studies; vibrational calculations and scaling study; characterization details of prepared Cu-CHA zeolite; potential reaction-energy landscape for all three active sites; and binding energy study of HONO species (PDF)

## Abbreviations

SCR: selective catalytic reduction
QM/MM: quantum mechanical/molecular mechanical
DFT: density functional theory
CHA: chabazite
DRIFTS: Diffuse Reflectance Infrared Fourier-Transform Spectroscopy
FTIR: Fourier transform infrared
MD: molecular dynamics
AIMD: *ab initio* molecular dynamics
PXRD: powder X-ray diffraction
EDX: energy-dispersive X-ray
BET: Brunauer–Emmett–Teller
MES: modulation excitation spectroscopy
PSD: phase-sensitive detection
