# Supplementary material for: Influence of Solvent on Selective Catalytic Reduction of Nitrogen Oxides with Ammonia over Cu-CHA Zeolite
Source: J Am Chem Soc. 2022 Dec 22;145(1):247–59. doi: 10.1021/jacs.2c09823 (PMC9837844; doi:10.1021/jacs.2c09823)
Supplement: Supplementary file 1 — ja2c09823_si_001.pdf [file ja2c09823_si_001.pdf]

Supporting Information

Influence of Solvent on Selective Catalytic Reduction of Nitrogen Oxides with Ammonia over Cu-CHA Zeolite

Jamal Abdul Nasir [1*], Jingcheng Guan [1], Thomas W. Keal [4], Alec W. Desmoutier[1], You Lu [4], Andrew M. Beale [1,5], C. Richard A. Catlow [1,2,3*], Alexey A. Sokol [1*].

1 Department of Chemistry, Kathleen Lonsdale Materials Chemistry, University College London, 20 Gordon Street, London WC1H 0AJ, United Kingdom
2 UK Catalysis Hub, Research Complex at Harwell, Rutherford Appleton Laboratory, R92 Harwell, Oxfordshire OX11 0FA, United Kingdom
3 School of Chemistry, Cardiff University, Park Place, Cardiff, CF10 3AT, United Kingdom
4 Scientific Computing Department, STFC Daresbury Laboratory, Keckwick Lane, Daresbury, Warrington, WA4 4AD, United Kingdom.
5 Department of Chemistry, Christopher Ingold Building, University College London, 20 Gordon Street, London WC1H 0AJ, United Kingdom

Email: jamal.nasir.18@ucl.ac.uk

Email: c.r.a.catlow@ucl.ac.uk

Email: a.sokol@ucl.ac.uk

Table S1. Selected calculated parameters of the optimized silica CHA chabazite, using two different DFT functionals, and their comparison with the experimental data.

Bond	Si-O distance [Å]			Bond	Si-O-Si angle [°]		
**Si-O**	**B97-2**	**BB1K**	**Exp.** [a]		**B97-2**	**BB1K**	**Exp.** (Å) [a]
**Si_1-O_1**	1.603	1.602	1.609	Si_1-O_1-Si_2	127.049	127.230	147.752
**Si_1-O_2**	1.588	1.588	1.609	Si_1-O_2-Si_3	166.093	166.157	150.131
**Si_1-O_3**	1.595	1.594	1.609	Si_1-O_3-Si_4	141.733	141.901	149.304
**Si_1-O_4**	1.592	1.592	1.610	Si_1-O_4-Si_5	163.665	163.774	149.435
**average**	**1.595**	**1.594**	**1.609**	**average**	**149.635**	**149.765**	**149.155**

[a] **Experimental data are taken from ref.** [1]

Metal incorporation in zeolite has been an important subject of investigation in heterogeneous catalysis. In particular, the Cu-doped CHA has been considered an essential material used for several important reactions, especially NH_3-SCR catalysis [2]. Commensurate with this study, we have proposed several catalytic sites for Cu^{2+} locations around Al-tetrahedra. To perform this study, we used the B97-2 functional and triple-ζ basis set def2-TZVP. The purpose of the study was to examine the possible configurations of the Cu^{2+}-OH species in CHA; their relative stability and the structural parameters (as compared to the experimental one). Although, the exact position of Cu ions and the active sites in Cu-CHA are still controversial [3]. It is also reported that in the presence of the adsorbate, the Cu ion relocates its position from 6MR to 8MR, an observation that comes from the formation of Cu^{2+}-NO_3 intermediates, where the energy reported in the 8MR is lower than in 6MR [8b]. The geometric parameters obtained with DFT calculations using ChemShell are in excellent agreement with the literature [4], and therefore, can be used to investigate the NH_3-SCR on Cu-CHA reaction.

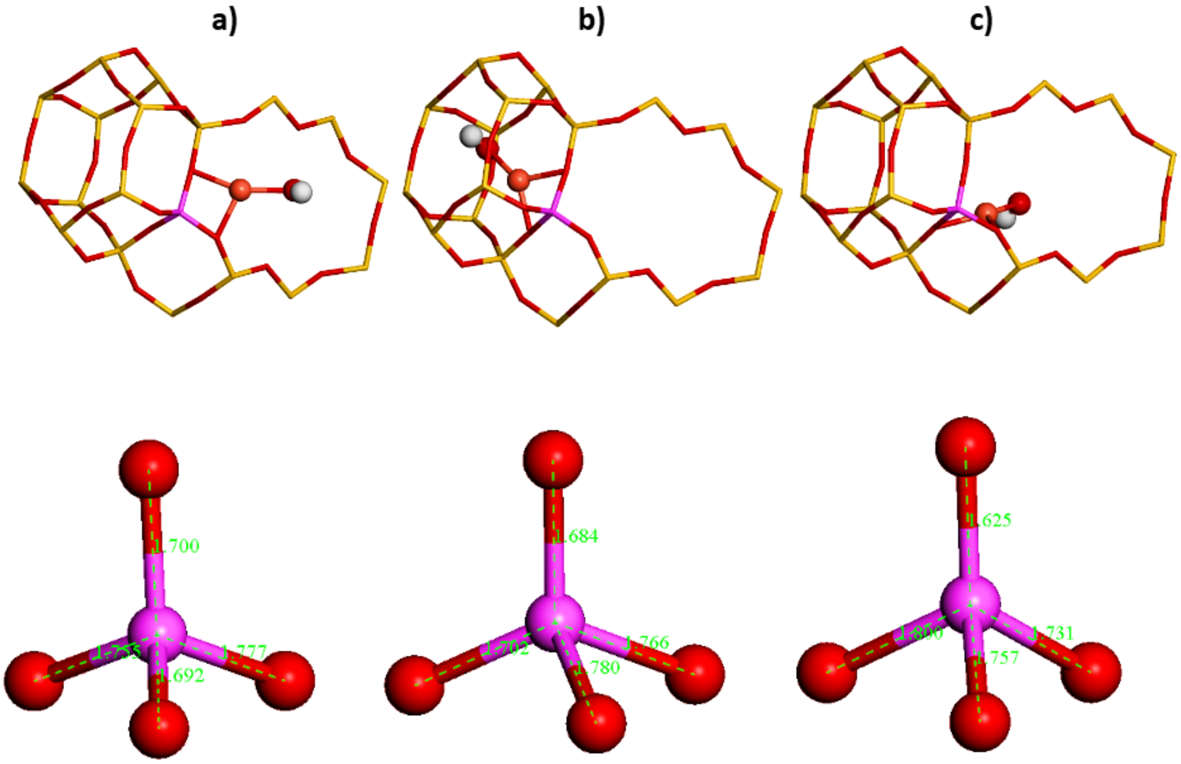

Figure S1. Representative Cu^{2+}-OH^{-} CHA Al-tetrahedra showing various possible locations of Cu^{2+}-OH^{-} within the CHA framework. Color code: Al, purple; Si, yellow; O, red; Cu, gray; N, blue; and H, white;

Table S2. The calculated bond lengths of the Al-O (Å) Cu^{2+}-OH^{-} CHA chabazite for the possible three configurations.

**Brønsted acid sites**	**Cu-a (Å)**	**Cu-b (Å)**	**Cu-c (Å)**	**Exp. (Å)** [a]
**Al-O1**	1.70	1.68	1.62	
**Al-O1**	1.75	1.78	1.75	
**Al-O1**	1.77	1.70	1.80	
**Al-O1**	1.69	1.76	1.73	
**Avg.**	**1.72**	**1.73**	**1.72**	**1.73**

[a]Experimental data are taken from ref. [1]

Effect of DFT functional and basis sets

To explore the effect of various simulation parameters on our results, we have tested different DFT functionals such as B97-2[5], B97-3[6], and BB1K[7] on a series of def2 basis sets[8] (Def2-SVP, Def2-TZVP, Def2-QZVP, Def2-TZVPD, Def2-QZVPD). In addition, we also tested different levels of theory such as CCSD(T) and CR-CCSD(T) to gauge the accuracy of the result relative to the experimental values as shown in Table S3.

Table S3. The calculated reaction energies (eV) for NO-activation reaction; both theoretical and experimental values. All theoretical calculations are performed using def2 basis sets [8].

**DFT**	**Def2-SVP**	**Def2-TZVP**	**Def2-QZVP**	**Def2-TZVPD**	**Def2-QZVPD**
**B97-2**	-7.817	-8.366	-8.421	-8.406	-8.458
**B97-3**	-7.865	-8.352	-8.455	-8.399	-8.481
**Bb1k**	-7.816	-8.309	-8.385	-8.350	-8.410
**Exp.**	-9.01				
**Post-HF**	**Def2-SVP**	**Def2-TZVP**	**Def2-QZVP**	**Def2-TZVPD**	**Def2-QZVPD**
**CCSD(T)**	-9.567	-9.925	-9.435	-9.124	-9.475
**CR-CCSD(T)**	-9.534	-9.0232	-9.370	-9.329	-9.849

$$3NO + 2NH_3 + O_2\ (Reactants) \rightarrow\ 2N_2\ + 3H_2O +\ NO_2(Products). \qquad (1)$$

Experimental $\Delta_f H°_0$ = -9.01 eV (-869 kJ/mol)

Theoretical = -8.37 eV

Table S4. The calculated reaction energies for reaction (1) are based on the standard enthalpy of formation of the species involved.

**Temperature (°C)**	**Reactants**	**Products**	**($\Delta_f H°$) kJ/mol**	**($\Delta_f H°$) eV**
**298**	182.02	-692.278	-874.298	-9.06
**0**	236.811	-632.208	-869.019	-9.01

Table S5. The standard enthalpy of formation values of the species at temperatures 298 °C and 0 °C.

Species	$\Delta_f H°$ (298.15 K)/kJ.mol^{-1}	$H°$ (298.15 K) - $H°$ (0)/kJ.mol^{-1}	$H°$ (0)
**NO**	91.3	8.675	99.975
**NH_3**	-45.94	10.043	-35.897
**NO_2**	33.2	13.015	46.215
**H_2O**	-241.826	9.905	-231.921
**N_2**	0	8.67	8.67
**O_2**	0	8.68	8.68

Standard enthalpy of formation $\Delta_f H°$ at 298.15 K, the quantity $H°$ (298.15 K) - $H°$ (0). Note that the value of 0 in the $\Delta_f H°$ column for an element indicates the reference state for that element.

Table S6. Calculated N_2 dissociation energies (eV); both theoretical and experimental values. All theoretical calculations are performed using def2 basis sets [8].

DFT	Def2-SVP	Def2-TZVP	Def2-QZVP	Def2-TZVPD	Def2-QZVPD
**B97-2**	9.839	9.885	9.890	9.896	9.905
**B97-3**	9.854	9.917	9.926	9.925	9.936
**Bb1k**	9.344	9.404	9.412	9.413	9.423
**Exp**	9.80				

$$N_2 \rightarrow 2N \qquad (2)$$

Experimental $\Delta_f H°_0$ = 9.80 eV (945 kJ/mol)

Theoretical = 9.88 eV

Vibrational calculation

To perform the vibrational study, we start by determining the vibrational frequencies by solving the eigenvalue problem as shown in Eq. 3, based on the harmonic approximation of the potential energy surface.

$$DL = \Lambda L. \qquad (3)$$

where D represents the dynamical matrix containing second-order derivatives of QM/MM energies with respect to nuclear displacements around a configurational equilibrium in mass-weighted Cartesian space. Λ and L are the diagonal matrix of eigenvalues and the eigenvectors-composed matrix, respectively. The eigenvectors are the normal modes of the system, and L is used as the transformation matrix whose columns are exactly the eigenvectors of the dynamical matrix D, transferring nuclear displacements in mass-weighted Cartesian coordinates to normal modes coordinates. Vibrational frequencies of normal modes are taken as the square roots of eigenvalues in the diagonal matrix Λ.

A drawback of a standard normal mode analysis is that the modes may be delocalized throughout the vibrational system. In the Zeolite QM/MM cluster, a normal mode involves delocalised combinations of vibrational motions from different chemical groups, for example, nitrate and nitrosamine groups, for which only fractions can be resolved in experimental vibrational spectra. To overcome the delocalisation problem, we analyse the vibrational frequencies along with localised modes by first transforming the normal modes into a set of localised vibrations of individual species [9]. Here in the case of the Chabazite structure, a local mode will in general be dominated by vibrations of single species. Of course, localised modes contribute to similar signatures in experimental spectra with close frequencies on homologous species, but they also allow us to learn much more accurately about the species with different surroundings, for example, solvent and ammonia in the silicious environment, and their impacts on the vibrations of active sites. To localize the modes we follow the method introduced by Jacob and Reiher[9] that applies unitary transformation repetitively, the so-called Jacobi sweeps procedure, to either maximise the atomic contributions in individual modes, by taking fractions of kinetic energies of atomic species in normal modes or to achieve the largest separations between centres of any pair of individual modes (Eq. 4-6). Since only a pair of modes have been involved in a unitary rotation during the localisation procedure, the orthogonality is maintained. In our work, we choose the latter criterion according to a previous report[10] that the localisation is proceeded using the maximal separations among modes. Once the unitary transformation is no longer able to increase the separations, the local modes are obtained.

$$\tilde{C}_{ip}^{sub} = \sum_{a=x,y,z} \left(\tilde{Q}_{i\alpha,p}^{sub}\right)^2. \qquad (4)$$

$$R_p^{center} = \sum_{i=1}^{n} \tilde{C}_{ip}^{sub} \, R_i \, . \qquad (5)$$

$$\xi_{dist}\left(\tilde{Q}^{sub}\right) = \sum_{p=1}^{k} (R_p^{center})^2 = \sum_{p=1}^{k} (\sum_{i=1}^{n} \tilde{C}_{ip}^{sub} \, R_i)^2. \qquad (6)$$

where $\xi_{dist}\left(\tilde{Q}^{sub}\right)$ is a function that quantifies how a localised set of modes is, and R_i is the Cartesian component of nucleus *i*.

Validation of Scaling factor: The computed quantum chemical harmonic vibrational frequencies (ν) are typically greater than the fundamentals ($\nu^{\sim}$) observed experimentally, and, therefore, are often scaled by empirical factors, to bring it closer to the "true" fundamental frequency. However, it is also known that it is impossible to describe well all bands of the complex diverse system using just one scaling factor, which can be related to different behaviour of different chemical bonds (cf. single bonds vs double bonds vs triple bonds). The reliability of our scaled vibrational frequencies is demonstrated by comparing them with the experimentally measured DRIFTS vibrational spectra, where all major bands are in very good agreement. In particular, we have used different scaling factors obtained from the comparison of the corresponding gas phase experimental and calculated values, for which we carefully chose reference molecules that are very close in structure and bonding to the corresponding intermediate species. For example, in the case of bidentate nitrate species with a double N-O_{str} bond, we calculated the scaling factor value based on the theoretical and experimental values for HNO_3 as a reference molecule, whereas for a resonance N-O_{str} bond (single to double) in nitrosamine species, we chose an average of NO fractions from similarly bonded NO_2 and N_2O molecules – see figure below.

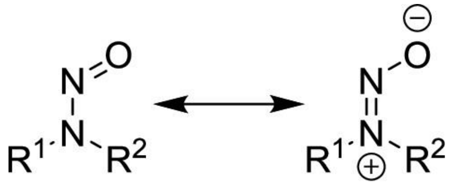

Resonating structure of nitrosamine species

Scaling factor and vibrational frequencies for nitrate species

Table S7. Calculated scaling factor value based on the theoretical and experimental vibrational frequencies (cm^{-1}) for $N\text{-}O_{str}$ of HNO_3 as a reference molecule.

**Theoretical (cm^{-1})**	3775	1814	1389	1342	936	809	677	603	492
**Experimental (cm^{-1})**	3550	1710	1331	1325	879	647	579	762	456
**Scaling factor**		0.943							

Table S8. Scaled vibrational frequencies for the bare nitrate species.

Assignments	Frequencies (cm^{-1})	Scale factor	Freq. after scaling(cm^{-1})
**1st N-O stretch**	1718	0.943	1620
**2nd N-O stretch**	1243	0.943	1172
**3rd N-O stretch**	1033	0.943	973
**4th N-O stretch**	830	0.943	782

Table S9. Scaled vibrational frequencies for the physisorbed water nitrate.

Assignments	Frequencies (cm^{-1})	Scale factor	Freq. after scaling (cm^{-1})
**1st N-O stretch**	1707	0.943	1609
**2nd N-O stretch**	1265	0.943	1192
**3rd N-O stretch**	1058	0.943	997
**4th N-O stretch**	834	0.943	786

Table S10. Scaled vibrational frequencies for the physisorbed ammonia nitrate.

Assignments	Frequencies (cm^{-1})	Scale factor	Freq. after scaling (cm^{-1})
**1st N-O stretch**	1697	0.943	1600
**2nd N-O stretch**	1275	0.943	1202
**3rd N-O stretch**	1057	0.943	996
**4th N-O stretch**	838	0.943	790

Scaling factor and vibrational frequencies for nitrosamine (Cu–N(=O)–NH_2) species

Table S11. Calculated scaling factor value based on the theoretical and experimental vibrational frequencies (cm^{-1}) for N-O_{str} of NO_2 as reference molecules.

**Theoretical (cm^{-1})**	1769	1432	781
**Experimental (cm^{-1})**	1618	1318	750
**Scaling factor**	0.915	0.92	

Table S12. Calculated scaling factor value based on the theoretical and experimental vibrational frequencies (cm^{-1}) for N-O_{str} of N_2O as reference molecules.

**Theoretical (cm^{-1})**	2401	1372	637
**Experimental (cm^{-1})**	2230	1250	600
**Scaling factor**		0.91	

Average = 0.915 + 0.92 + 0.91/3 = **0.915** (average of NO fractions from NO_2 and N_2O molecules).

Table S13. Calculated scaling factor value based on the theoretical and experimental vibrational frequencies (cm^{-1}) for N-N_{str} of N_2O as reference molecules.

**Theoretical (cm^{-1})**	2401	1372	637
**Experimental (cm^{-1})**	2230	1250	600
**Scaling factor**	0.929		

Table S14. Calculated scaling factor value based on the theoretical and experimental vibrational frequencies (cm^{-1}) for N-N_{str} of N_2H_2 as reference molecules.

Theoretical (cm^{-1})	3283	3252	1696	1686	1363	1360
Experimental (cm^{-1})	3128	3120	1583	1529	1286	1350
Scaling factor				0.907		

Average = 0.907+0.929/2 = **0.918** (average of N-N fractions from N_2H_2 and N_2O molecules.

Table S15. Scaled vibrational frequencies (cm^{-1}) for the bare nitrosamine species.

Assignments	Frequencies (cm^{-1})	Scale factor	Freq. after scaling (cm^{-1})
N-O stretch	1615	0.915	1478
N-N stretch	1225	0.918	1124

Table S16. Scaled vibrational frequencies for the physisorbed water nitrosamine species.

Assignments	Frequencies (cm^{-1})	Scale factor	Freq. after scaling
N-O stretch	1603	0.915	1467
N-N stretch	1224	0.918	1123

Table S17. Scaled vibrational frequencies for the physisorbed ammonia nitrosamine species.

Assignments	Frequencies (cm^{-1})	Scale factor	Freq. after scaling(cm^{-1})
N-O stretch	1597	0.915	1462
N-N stretch	1210	0.918	1111

Table S18. Calculated scaling factor values based on the theoretical and experimental vibrational frequencies of N-H_{str}, N-H_{sciss} and N-H_{wagg} of NH_3 as a reference molecule.

Experimental (cm^{-1})	1626 (cm^{-1})	3443 (cm^{-1})	932 (cm^{-1})
Calculated (cm^{-1})	1681 (cm^{-1})	3637 (cm^{-1})	1044 (cm^{-1})
Scaling	0.967 (scissors)	0.947 (Strech)	0.893 (Wagg)

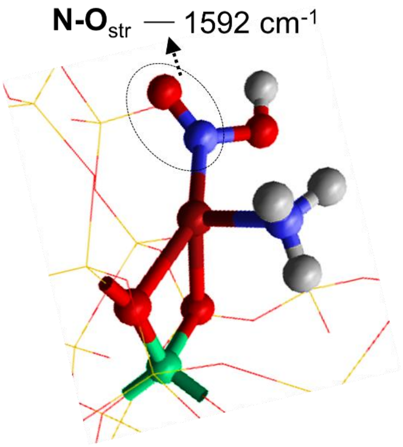

Figure S2. Vibrational spectroscopic analysis of the Cu-(N(=O)-OH)-NH_3 species.

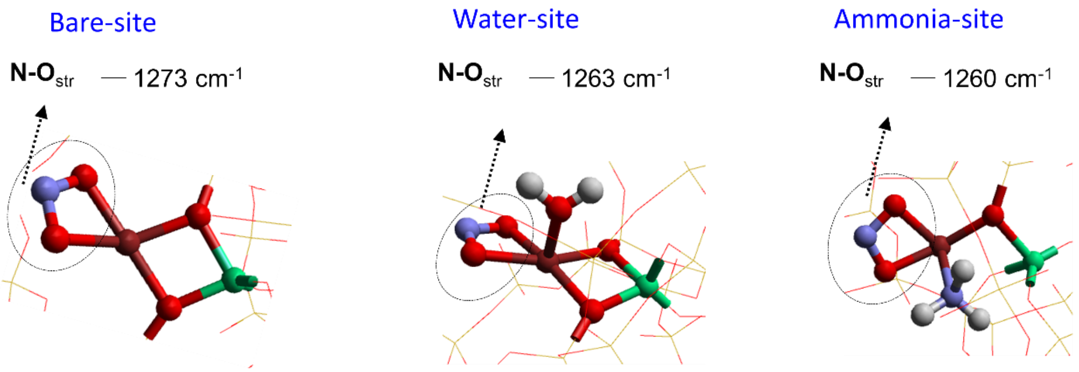

Figure S3. Vibrational spectroscopic analysis of the N=O$_{str}$ vibrational mode of bidentate-nitrite (Cu–NO_2) species for all three sites.

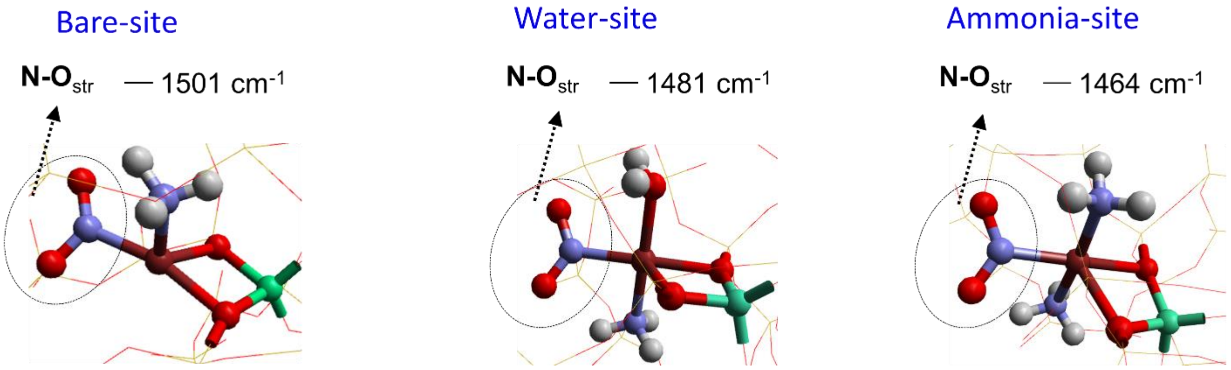

Figure S4. Vibrational spectroscopic analysis of the N=O$_{str}$ vibrational mode of bidentate-nitrite (Cu–(NO_2)–NH_3) species for all three sites.

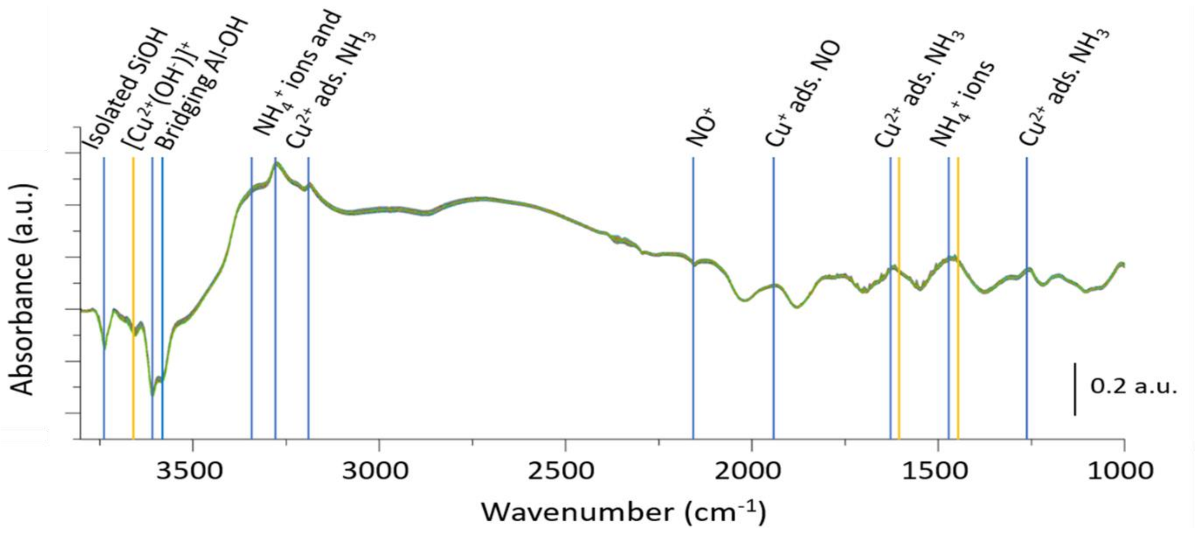

Figure S5. DRIFTS spectra collected during the NO concentration modulation experiment.

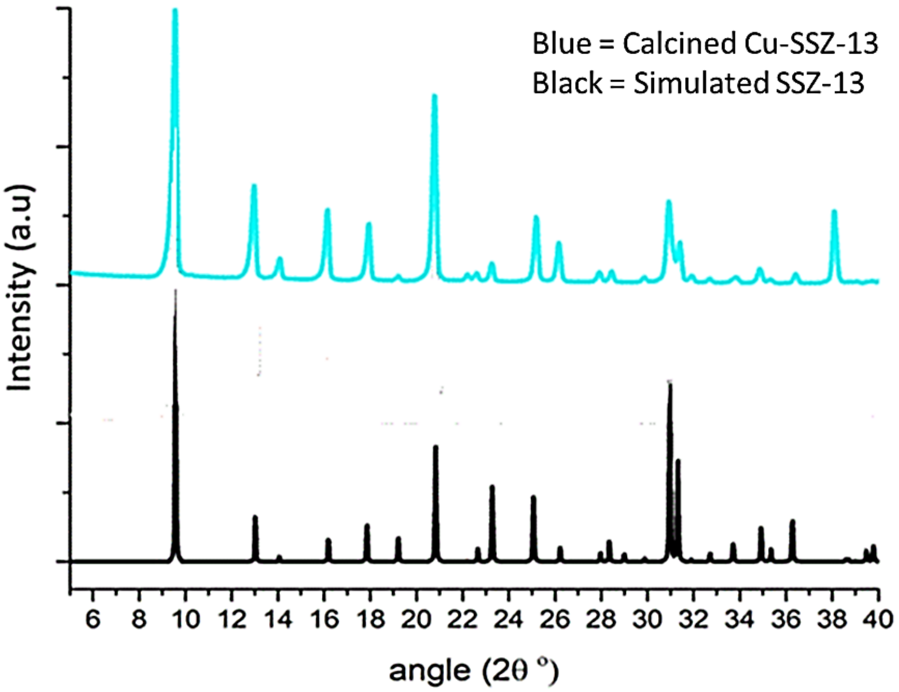

Figure S6: **Diffraction patterns**: Calcined Cu-SSZ-13 (blue), simulated PXRD (black), collected at room temperature in a hydrated state. Note reflection at 38 ° is due to the Teflon sample holder.

Table S19: XRF element composition of Cu-SSZ-13 sample.

Element	Wt.%	Wt.% Sigma
**O**	53.12	0.15
**Al**	3.19	0.04
**Si**	40.77	0.13
**Cu**	2.92	0.10
**Total:**	100.00	

Table S20: Surface area and pore volume of Cu-SSZ-13 sample.

Sample	Micropore volume (cc/g)	Micropore area (m^2/g)
**Cu-SSZ-13**	0.28	758

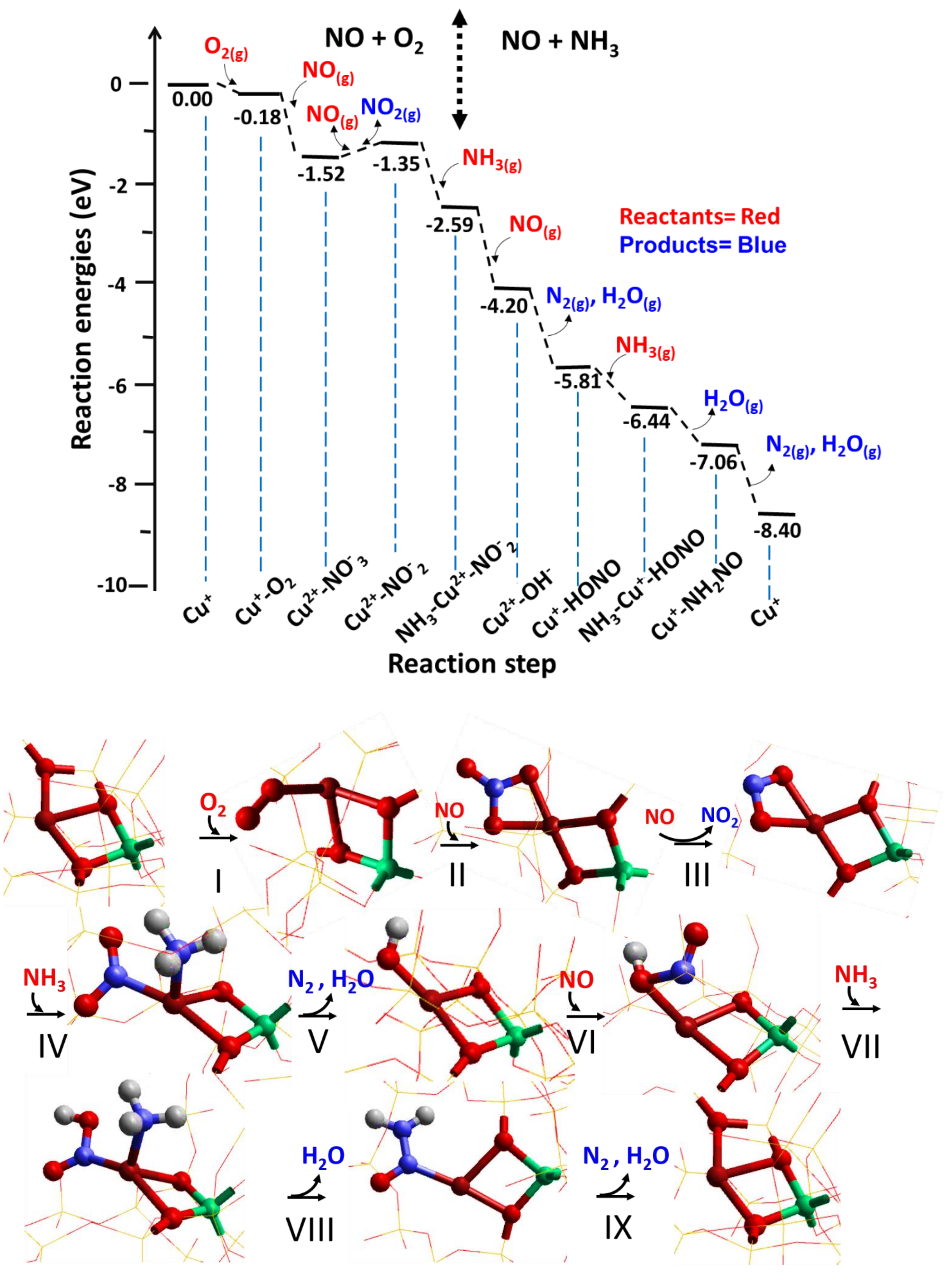

Figure S7. The potential reaction-energy landscape for NH_3-SCR on activated bare Cu-CHA site. The structures of each corresponding intermediate species residing on bare Cu-CHA are presented in the lower panel. The model used is shown as an extra framework**. Atom colour codes**: Cu (brown), Al (green), O (red), N (blue), and H (white). The framework SiO_2 is shown using a wire framework motif.

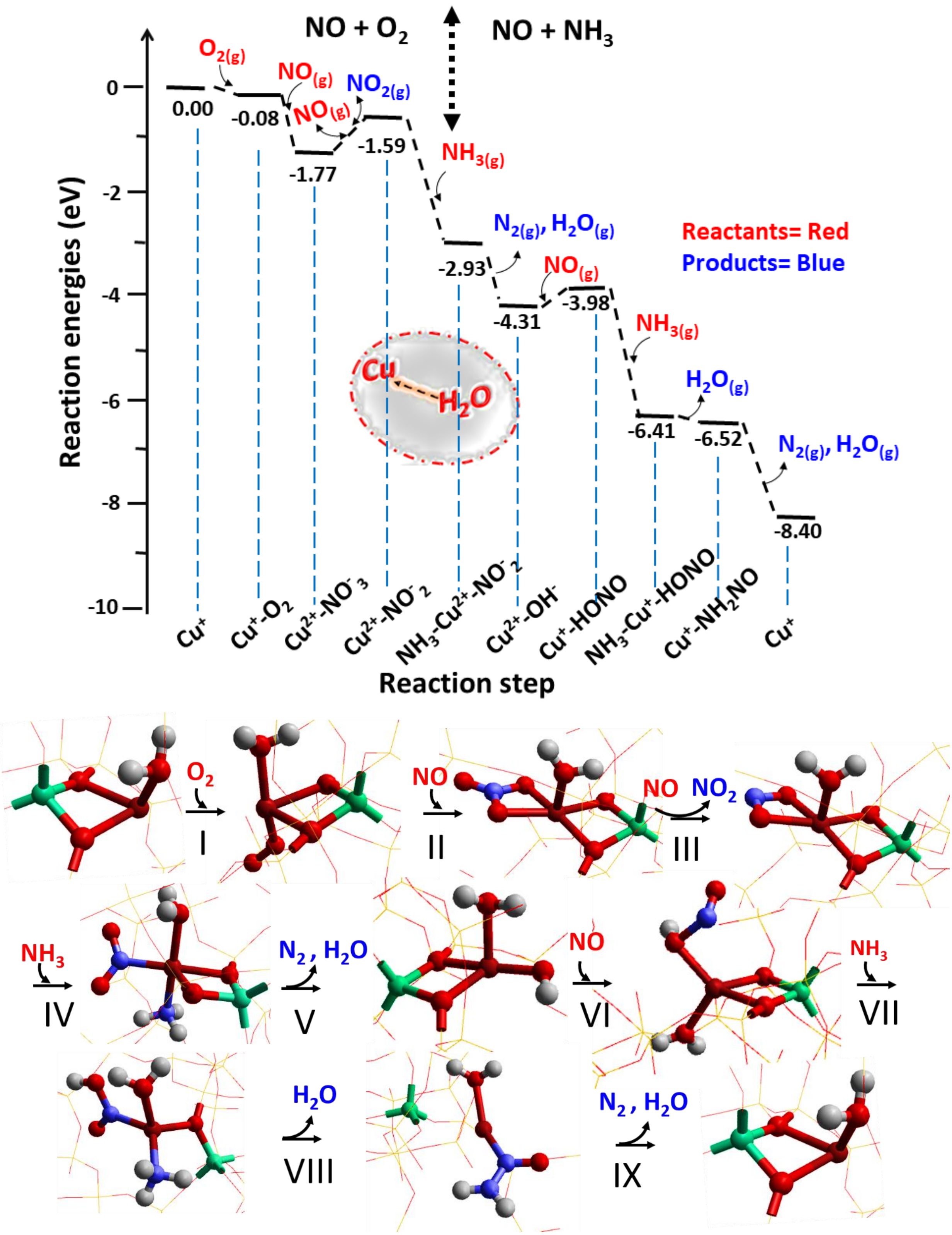

Figure S8. The potential reaction-energy landscape for NH_3-SCR on activated physisorbed water Cu-CHA site. The structures of each corresponding intermediate species residing on physisorbed water Cu-CHA are presented in the lower panel. The model used is shown as an extra framework**. Atom colour codes**: Cu (brown), Al (green), O (red), N (blue), and H (white). The framework SiO_2 is shown using a wire framework motif.

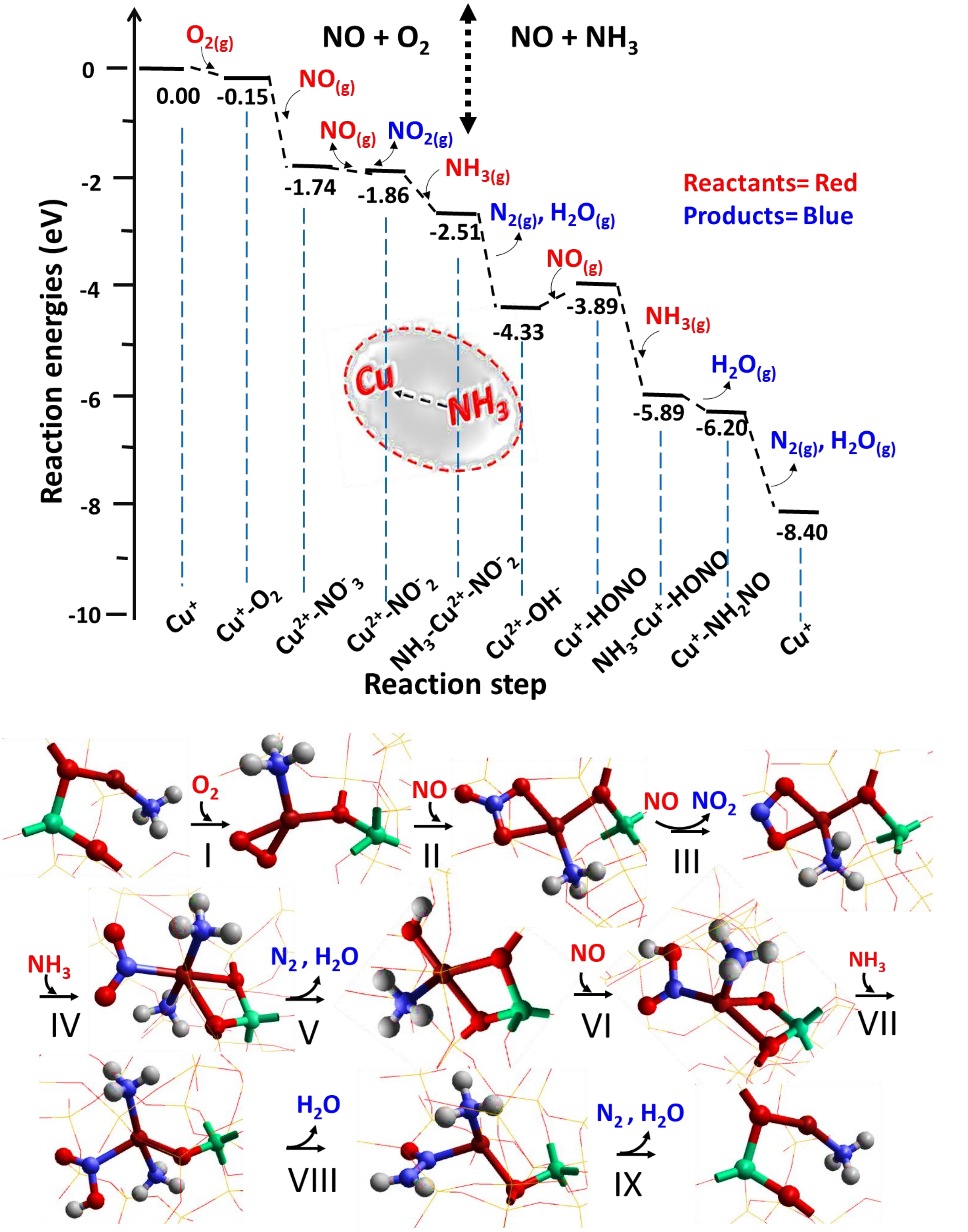

Figure S9. The potential reaction-energy landscape for NH_3-SCR on activated physisorbed ammonia Cu-CHA site. The structures of each corresponding intermediate species residing on physisorbed ammonia Cu-CHA are presented in the lower panel. The model used is shown as an extra framework**. Atom colour codes**: Cu (brown), Al (green), O (red), N (blue), and H (white). The framework SiO_2 is shown using a wire framework motif.

$$Cu{-}N({=}O){-}NH_2 \longrightarrow Cu^{+} + H_2O + N_2$$

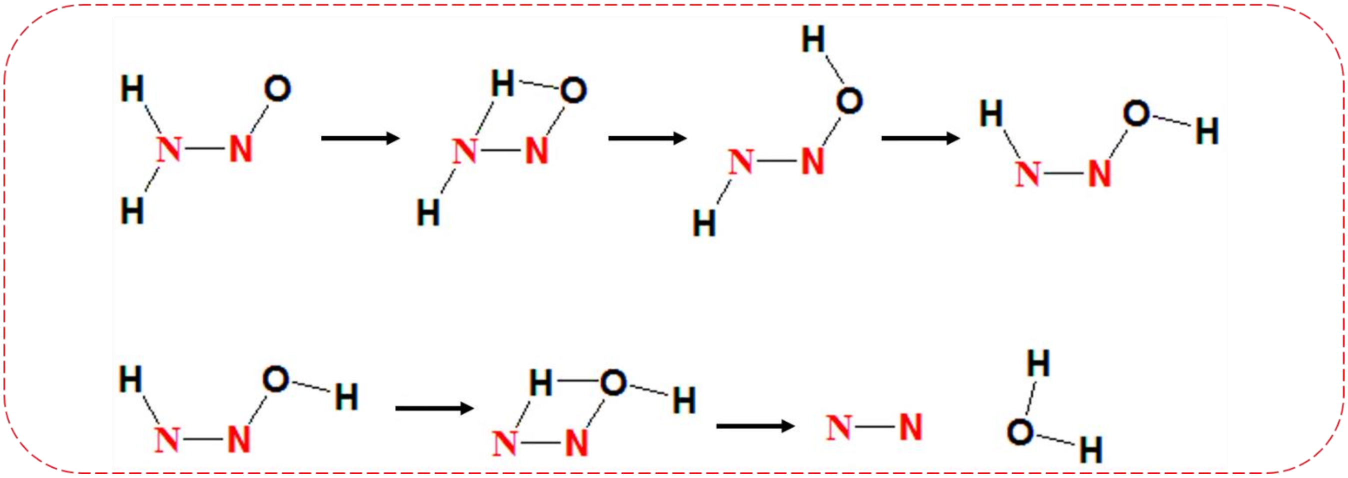

Figure S10. Schematic illustrations of isomeric decompositions of H_2NNO intermediates.

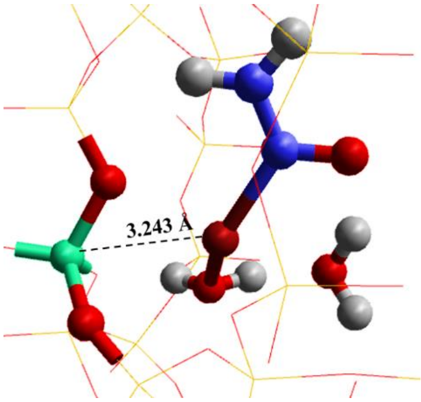

Figure S11. The effect of solvation (H_2O coordination) on H_2NNO intermediates. **Colour code:** Al, green; Si, yellow; O, red; Cu, brown; N, blue; and H, white;

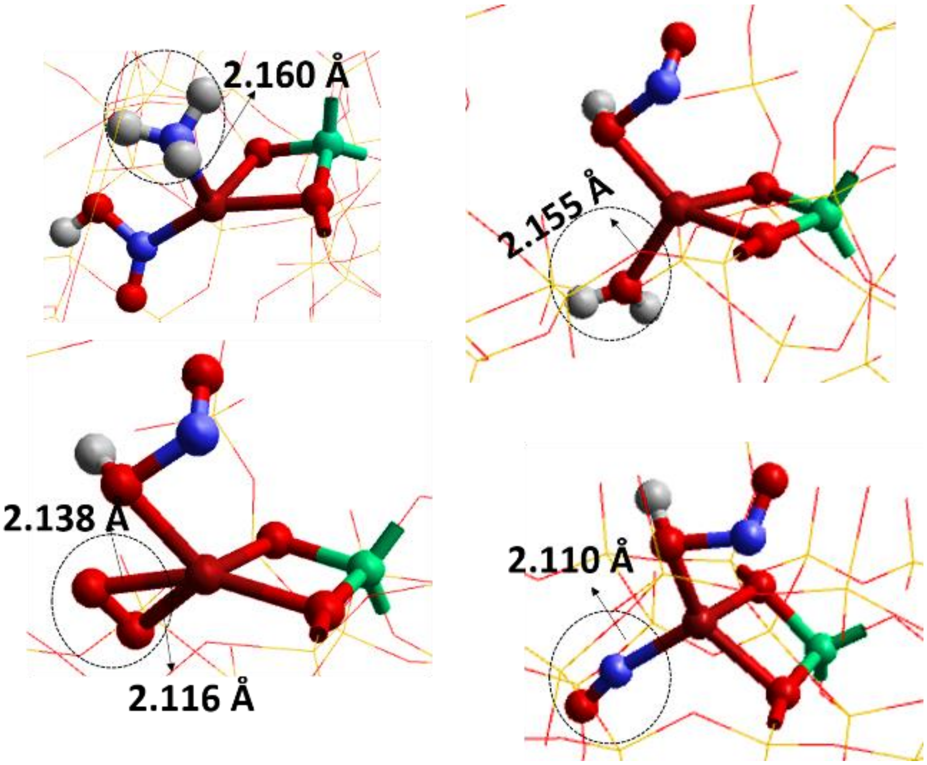

Figure S12. The binding of HONO species with NH_3, H_2O, O_2, and NO. **Colour code:** Al, green; Si, yellow; O, red; Cu, brown; N, blue; and H, white;

References

1. Smith, J.; Rinaldi, F.; Dent Glasser, L., Crystal structures with a chabazite framework. II. Hydrated Ca-chabazite at room temperature. *Acta Crystallographica* **1963,** *16* (1), 45-53.
2. Groothaert, M. H.; van Bokhoven, J. A.; Battiston, A. A.; Weckhuysen, B. M.; Schoonheydt, R. A., Bis (μ-oxo) dicopper in Cu-ZSM-5 and its role in the decomposition of NO: a combined in situ XAFS, UV− Vis− Near-IR, and kinetic study. *J. Am .Chem. Soc.* **2003,** *125* (25), 7629-7640.
3. Bates, S. A.; Verma, A. A.; Paolucci, C.; Parekh, A. A.; Anggara, T.; Yezerets, A.; Schneider, W. F.; Miller, J. T.; Delgass, W. N.; Ribeiro, F. H., Identification of the active Cu site in standard selective catalytic reduction with ammonia on Cu-SSZ-13. *J. catal.* **2014,** *312*, 87-97.
4. Eilertsen, E. A.; Arstad, B.; Svelle, S.; Lillerud, K. P., Single parameter synthesis of high silica CHA zeolites from fluoride media. *Micropor. Mesopor. Mat.* **2012,** *153*, 94-99.
5. Wilson, P. J.; Bradley, T. J.; Tozer, D. J., Hybrid exchange-correlation functional determined from thermochemical data and ab initio potentials. *The Journal of Chemical Physics* **2001,** *115* (20), 9233-9242.
6. Keal, T. W.; Tozer, D. J., Semiempirical hybrid functional with improved performance in an extensive chemical assessment. *The Journal of chemical physics* **2005,** *123* (12), 121103.
7. Zhao, Y.; Lynch, B. J.; Truhlar, D. G., Development and assessment of a new hybrid density functional model for thermochemical kinetics. *The Journal of Physical Chemistry A* **2004,** *108* (14), 2715-2719.
8. Zheng, J.; Xu, X.; Truhlar, D. G., Minimally augmented Karlsruhe basis sets. *Theoretical Chemistry Accounts* **2011,** *128* (3), 295-305.
9. Jacob, C. R.; Reiher, M., Localizing normal modes in large molecules. *The Journal of chemical physics* **2009,** *130* (8), 084106.
10. Cheng, X.; Steele, R. P., Efficient anharmonic vibrational spectroscopy for large molecules using local-mode coordinates. *The Journal of Chemical Physics* **2014,** *141* (10), 104105.
